# Internal Validation of a Machine Learning-Based CDSS for Antimicrobial Stewardship

**DOI:** 10.3390/life15071123

**Published:** 2025-07-17

**Authors:** Ari Frenkel, Alicia Rendon, Carlos Chavez-Lencinas, Juan Carlos Gomez De la Torre, Jen MacDermott, Collen Gross, Stephanie Allman, Sheri Lundblad, Ivanna Zavala, Dave Gross, Jessica Siegel, Soojung Choi, Miguel Hueda-Zavaleta

**Affiliations:** 1 Arkstone Medical Solutions, Boca Raton, FL 33428, USA; afrenkel@arkstonemedical.com (A.F.); arendon@arkstonemedical.com (A.R.); jgomez@labroe.com (J.C.G.D.l.T.); jmacdermott@arkstonemedical.com (J.M.); cgross@arkstonemedical.com (C.G.); sallman@arkstonemedical.com (S.A.); slundblad@arkstonemedical.com (S.L.); izavala@arkstonemedical.com (I.Z.); dave.gross@arkstonemedical.com (D.G.); jessicasiegel6701@gmail.com (J.S.); soojungchoi2018@health.fau.edu (S.C.); 2 Hospital Nacional Edgardo Rebagliati Martins, Lima 15073, Peru; cchavezl1@unmsm.edu.pe; 3 Facultad de Medicina, Universidad Nacional Mayor de San Marcos, Lima 15072, Peru; 4 Clinical Laboratory Roe, Lima 15076, Peru; 5 Diagnóstico, Tratamiento e Investigación de Enfermedades Infecciosas y Tropicales, Universidad Privada de Tacna, Tacna 23003, Peru

**Keywords:** machine learning, antimicrobial stewardship, antibiotic resistance, clinical decision support

## Abstract

**Background:** Antimicrobial stewardship programs (ASPs) are essential in combating antimicrobial resistance (AMR); however, limited resources hinder their implementation. Arkstone, a biotechnology company, developed a machine learning (ML)-driven clinical decision support system (CDSS) to guide antimicrobial prescribing. While AI (artificial intelligence) applications are increasingly used, each model must be carefully validated. **Methods:** Three components of the ML system were assessed: (1) A prospective observational study tested its ability to distinguish trained from novel data using various validation techniques and BioFire molecular panel inputs. (2) An anonymous retrospective analysis of internal infectious disease lab results evaluated the recognition of novel versus trained complex datasets. (3) A prospective observational validation study reviewed clinical recommendations against standard guidelines by independent clinicians. **Results:** The system achieved 100% accuracy (F1 = 1) in identifying 111 unique novel data points across 1110 tests over nine training sessions. It correctly identified all 519 fully trained and 644 novel complex datasets. Among 644 clinician-trained reports, there were no major discrepancies in recommendations, with only 100 showing minor differences. **Conclusions:** This novel ML system demonstrated high accuracy in distinguishing trained from novel data and produced recommendations consistent with clinical guidelines. These results support its value in strengthening CDSS and ASP efforts.

## 1. Introduction

Machine learning (ML), a subset of artificial intelligence (AI), has rapidly transformed diverse sectors, including healthcare, by enabling systems to learn from data, identify patterns, and make decisions with minimal human intervention [1,2]. In clinical settings, ML-based clinical decision support systems (CDSSs) are increasingly used to aid in diagnosis, treatment planning, and antimicrobial stewardship [3,4,5]. A critical element in the deployment of these ML models is the rigorous validation process, which ensures the model’s reliability, generalizability, and accuracy when presented with new and complex data. This process is important, as models trained on limited data can overfit, capturing noise rather than relevant patterns, leading to poor performance when exposed to new data [6]. Furthermore, a lack of structured data review processes in some ML systems raises concerns about the accuracy of their recommendations, particularly in healthcare settings where critical variables might not be included in the analysis [7].

The potential of ML-driven CDSSs to improve patient care and public health is considerable, yet there are significant barriers to widespread adoption; one of these is the lack of comprehensive validation techniques [8] and confidence in an accurate working model. As previous attempts to implement ML-based systems in healthcare have shown, challenges such as poor data integration, concerns about data privacy, limited clinical applicability, and inaccurate recommendations have led to the discontinuation of various clinical support systems such as IBM Watson for Oncology [9,10,11] and DeepMind Health’s Streams [12,13,14]. These experiences highlight the need for robust, ethical, and clinically focused validation approaches to ensure the safe and effective integration of ML into clinical practice [15]. In addition, due to the rapid pace of evolving technology, formal validation methods are lacking, with limited data on the ideal validation process and evidence that ML processes are accurate [16]. Lastly, because of the uniqueness of the ML model in this study, to our knowledge, there are no studies evaluating its capabilities and its validation methods. Therefore, it is essential that we examine and test the capabilities of this system [17,18].

Arkstone, a biotechnology company, has developed a unique ML model for real-time, patient-specific infectious disease guidance integrated with laboratory results. This system provides clinicians with actionable recommendations aligned with clinical guidelines, intending to improve antimicrobial stewardship and reduce antibiotic overuse.

This study aimed to internally validate the system by evaluating its performance in training data and the system’s ability to recall the trained data and distinguish it from new data accurately. By analyzing the ML model, the integration of these tools into clinical practice can be achieved confidently. In addition, this study will evaluate the model’s robustness, accuracy, and generalizability for clinical use across diverse settings by evaluating the accuracy of the clinical recommendations themselves, which requires human input (human-in-the-loop (HITL) machine learning). This study aims to provide evidence to support the use of similar tools that enhance infectious disease knowledge and antimicrobial stewardship, particularly in resource-limited environments.

## 2. Materials and Methods

### 2.1. Model Description

Data enters the ML model via results sent by laboratories. This typically occurs in real time via an HL7 or API interface but can also be achieved through manual uploads. The data sent includes patient demographics, laboratory findings (organisms, antibiotic susceptibility, and resistance genes), sample sources, diagnostic codes, allergies, and pregnancy status. Within seconds to minutes, a concise, single-page PDF is generated that provides recommendations on the appropriate antimicrobial, if applicable (Supplement S1).

For scalable and effective processing of data, a unique machine learning model was developed that incorporates multiple validation techniques simultaneously, including applied K-fold cross-validation, random subsampling, and holdout validation (Supplement S2 and Supplement S3). The combination of methods, also called Antimicrobial Intelligence, is applied in real time (prospective validation), leveraging live data streams. A key component of this is the system’s inability to suggest new treatment options as well as to provide recommendations for untrained data sets. The system, therefore, relies on HITL processes on multiple levels to ensure the data is trained accurately. This step is critical at every stage of data training, ensuring that expert oversight is consistently applied. In addition to requiring human approval to train data, HITL is required and repeated by different infectious disease experts to ensure consistency and accuracy on how the data is trained, minimizing the risk of human error and bias. Furthermore, once the data is finally trained by multiple infectious disease experts, it does not stay in this status indefinitely. Data that has been rigorously trained previously gets pushed back into a status requiring it to be trained again, ensuring that data is repeatedly and periodically retrained so that information is up to date and error-free. This also allows for updates to medical recommendations that may have changed since initial training. This hybrid model that incorporates both human oversight and set algorithms ensures its adaptability to new and ever-evolving data.

**Figure 1:**
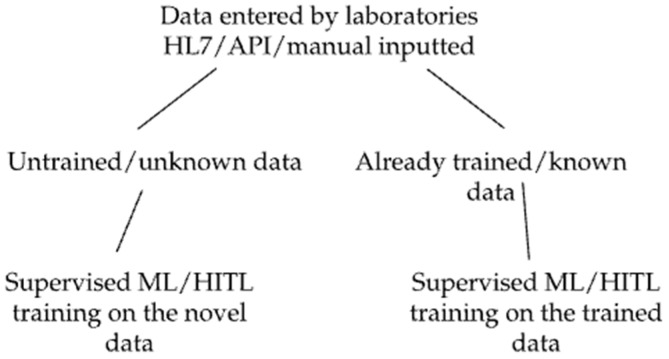
Simplified diagram of processes in Akstone machine learning.

The validation process is divided into three key elements that will be evaluated separately: (1) evaluation of the system’s ability to distinguish and recall trained from new single data points; (2) evaluation of the system’s ability to distinguish and recall trained from new complex data sets (Figure 1, where single data points refer to individual data points such as a gene, organism, allergy, or diagnosis, and complex data sets correspond to groups of single data points, such as those from a lab result that includes multiple data points combined together); and (3) evaluation of the accuracy of the clinical recommendations output by the system.

### 2.2. Data Sources and Preparation

Data were obtained from the Arkstone laboratory results database. The data set included positive and negative microbiology results, as well as demographic data such as patient age and sex, ICD-10-CM codes, allergies, pregnancy status, source of specimen, organism, sensitivity information, and resistance gene information. Diagnostic modalities included molecular or standard culture techniques. Prior to analysis, all data were de-identified according to HIPAA (Health Insurance Portability and Accountability Act of 1996) guidelines. Data were pre-processed to ensure consistency in format and coding.

### 2.3. Study Design

This validation study consisted of two components: a prospective observational phase and a retrospective analysis of real-world data from Arkstone’s database. This study was conducted in three sequential phases, each designed to evaluate different aspects of the machine learning system’s clinical performance. The research team accessed the data set remotely from their respective locations, without intervening in clinical care or altering the existing workflow of the system. Researchers reviewing reports were not the same individuals involved in formulating the initial clinical recommendations to avoid confirmation bias.

The primary objective of this study was to evaluate the internal validation process of the machine learning (ML) model through three critical components: (a) recognition of novel versus trained data: this component assessed the model’s ability to distinguish between previously unseen (untrained) data and data used during the model’s training process; (b) recognition of complex datasets: this involved evaluating the system’s performance in classifying large and heterogeneous datasets composed of multiple data points from various sources; and (c) human-in-the-loop (HITL) component: this assessed the accuracy of treatment recommendations generated by the system which requires human input via the HITL process.

### 2.4. Ethical Considerations and Data Availability

The study protocol was approved by the Institutional Ethics Committee of the Faculty of Health Sciences at the Private University of Tacna (FACSA-CEI/224-12-2024). All procedures complied with the Declaration of Helsinki and HIPAA guidelines. This study involved no risk to patients, and informed consent was not required, as all data were either retrospective, anonymized, or publicly available. The data set consisted of publicly accessible BioFire panel results and fully de-identified internal laboratory submissions. No protected health information (PHI) was accessed or disclosed (Supplement S2).

### 2.5. Procedures

#### 2.5.1. Element 1: Evaluation of the System’s Ability to Distinguish and Recall Trained from New Single Data Points

Element 1 involved a retrospective observational approach to evaluate the system’s ability to generate accurate recommendations based on individual data points, using multiple validation techniques. New data, also known as untrained data, refers to information the system has not previously encountered. Recognizing untrained data and subsequently training the system to identify it in future encounters are critical. Data input into the system was sourced from FDA-approved molecular diagnostic BioFire panels published on the BioFire website (https://www.biofiredx.com/products/the-filmarray-panels/ [accessed on 15 April 2025]). Panels do not contain any patient information but contain data regarding the type of panel, source of specimen, organism targets, and resistance gene targets. This was to ensure standardized nomenclature of panel types, organisms, and resistance genes. The new data encompassed six different standardized infectious disease panels (Table 1).

The data were selected based on the fact that these panels are among the few FDA-approved comprehensive molecular panels currently available. Additionally, the microbes and resistance markers tested by these panels are considered industry standard. Panel information was uploaded into the system and accessed remotely, from Boca Raton, and repeatedly analyzed on subsequent days.

To ensure the system recognized the uploaded data as novel and untrained, each data point was enclosed in brackets. The data set was then collectively input into the system (Table 2), which successfully identified it as entirely new, as bracketed data is never present in the system’s training inputs before. This approach helps prevent overfitting and bias and avoids introducing data already known to the model. Once the data was established as new and untrained, the data was then categorized according to each panel (Table 2) as designed by the panel manufacturer and entered into the system again. Here, too, the system identified all the variables entered into the system correctly as untrained data. This was expected since training did not occur between training sessions 1 and 2.

The panels were then randomized, and the data were split multiple times to form new data sets containing unique random variables (random subsampling). These new data sets were distributed into six groups: five of these groups became the folds for K-fold cross-validation (Table 2), and the sixth data set was used for holdout validation (Table 3). After training on one fold, the data sets from all six panels were re-entered into the system for analysis (Table 2). Once all the sets were analyzed, all the data were again introduced back into the system in its entirety for evaluation (Table 2).

#### 2.5.2. Element 2: Evaluation of the System’s Ability to Distinguish and Recall Trained from New Complex Datasets

An anonymous retrospective analysis was conducted using internal data submitted by various laboratories containing infectious disease results. Data within each lab result includes many variables sent by the lab, such as patient demographics, allergies, organisms, resistance genes, pregnancy status, diagnostic codes, and more. The research team accessed the data remotely from their respective locations. These results were randomly selected by choosing a random 24 h period (all results from Thursday, 1 August 2024, were used).

Patient-specific information was withheld from the researchers and was inaccessible. A team of three infectious disease specialists independently reviewed each laboratory result and evaluated the accuracy of how the system classified it (trained or untrained). Each result was reviewed by at least two members of the team, and discrepancies in classification were resolved through discussion until consensus was reached. The reviewers were not involved in the initial training of the data to avoid biases.

Data is considered untrained by the system if either a single variable is new or if a data set has a new combination of trained data (regardless of whether the individual data points have been trained). Fully trained data sets require not only each data point within the data set to be seen by the system, but also at least two complete training sessions involving the same data set configuration, in line with the criteria defined for “Auto-approve” status (Table 4). This means that the system must process the same combination of data points a minimum of two times before the data set is considered fully trained.

#### 2.5.3. Element 3: Evaluation of Human In-the-Loop Component and the Accuracy of Clinical Recommendations

A prospective evaluation of the recommendations generated by human-enhanced models (HITL) was conducted during the training process. The primary objective was to assess the appropriateness of recommendations that involved human intervention, which could lead to inaccurate recommendations. To assess this, the issued reports were evaluated by an independent team of infectious disease experts who reviewed them and determined, based on current clinical practice guidelines (IDSA, CDC) and recommendations from regulatory agencies (FDA, CLSI), and of course their own clinical experience and judgment, whether the issued reports showed major or minor discrepancies. This was assessed using a structured six-question questionnaire:

- Were the microbes being treated as pathogens accurately identified? [18]
- Does the antibiotic recommended in OneChoice have activity against the microbe that is presumed to be the pathogen?
- Was the recommended dose accurate?
- Was the recommended duration of treatment accurate?
- Was the preferred therapy the optimal therapy?
- Were there organisms that should have been addressed but were not?

Based on their responses, reviewers categorized discrepancies in HITL-trained outputs as either major or minor: (a) major discrepancies—failure to identify a pathogen that required treatment or recommending antibiotics that were ineffective against the identified microbe(s); (b) minor discrepancies—incorrect antibiotic dosage or treatment duration (outside FDA- or guideline-based ranges), a suboptimal choice when a better preferred or alternative therapy was available. This review process aimed to ensure the integrity of HITL-influenced recommendations and identify opportunities for further refinement of the system.

### 2.6. Data Analysis

Data analysis was performed using the STATA 17 statistical package.

For element 1: To evaluate the system’s ability to distinguish between trained vs. untrained data, the following metrics were calculated: (a) accuracy—proportion of data points correctly classified as trained or untrained; (b) precision—proportion of instances that were correctly identified as trained data; (c) recall—ability of the trained model to correctly identify previously trained data points; and (d) F1 score: the harmonic mean of precision and recall.

For element 2: To evaluate the system’s ability to distinguish between trained vs. untrained complex data sets, the following metrics were calculated: (a) true positive rate (TPR)—proportion of fully trained data sets correctly identified; (b) true negative rate (TNR)—proportion of untrained datasets correctly identified; (c) false positive rate (FPR)—proportion of untrained datasets incorrectly classified as trained; and (d) false negative rate (FNR)—proportion of fully trained datasets incorrectly classified as untrained.

For element 3: To evaluate the accuracy of HITL in providing accurate clinical recommendations, the percentage of reports with major and minor discrepancies was calculated.

## 3. Results

### 3.1. Element 1: Evaluation of the System’s Ability to Distinguish and Recall Trained from New Single Data Points

From the six panels (Table 1), there were initially 192 data points. However, after removing duplicate variables that spanned across multiple panels, 111 unique variables remained. Each panel was fully input into the system. Since this was novel data, the system correctly identified all these variables as new (Table 2).

The proportion of true positive results (correctly identified as trained) to the total predicted positives (all instances predicted as trained) is a measure of precision (Table 5). True positives were calculated by reintroducing previously trained data sets (used in the K-fold and holdout sessions) and evaluating whether the system recognized them correctly as trained. Similarly, the proportion of true positives to the total actual positives (all actual trained data points) is a measure of recall. This is collectively assessed using the F1 score.

The proportion of true positive results (i.e., correctly identified as trained) relative to the total number of instances predicted as trained represents the system’s precision (Table 6). Similarly, the ratio of true positives to all actual trained data points reflects the system’s recall. Together, these metrics demonstrate the system’s flawless performance in accurately distinguishing between trained and untrained data under the evaluated conditions.

### 3.2. Element 2: Evaluation of the System’s Ability to Distinguish and Recall Trained from New Complex Data Sets

**Figure 2:**
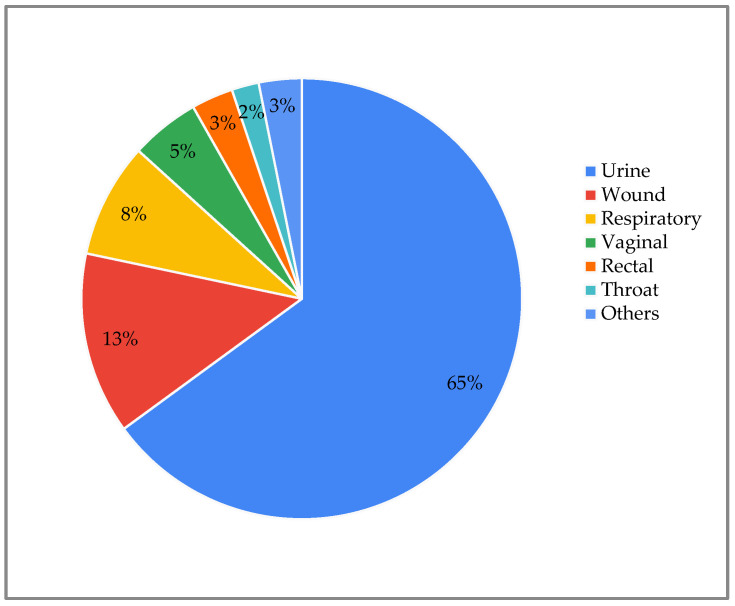
Types of samples submitted for analysis.

A total of 1401 real laboratory results were analyzed from the systems database (Supplement S2). These results were randomly selected by choosing a 24 h period at random. The data set was diverse, encompassing results from 66 laboratories located in 55 different regions across 24 states and 1 international site. This data included the following: 176 specified provider locations (12%), 936 unspecified practice locations (62%), 198 specified facility locations (13%), and 200 non-specified facility locations (13%). A total of 65% of the clinical samples analyzed were urine specimens, representing the most frequent sample type. This was followed by wound (13%), respiratory (8%), and vaginal samples (5%). Other less frequent sources included rectal (3%), throat (2%), nail (2%), oral (1%), urogenital, epidermal, and unknown sources (all <1%) (Figure 2).

Among the 1401 results analyzed, 238 (16.98%) were confirmed as true negatives—these were negative reports containing no relevant pathogen or antimicrobial concern and were correctly identified by the system as such (untrained). The remaining 1163 reports (83.02%) were classified as positive. Of these, 519 (44.62%) were identified by the system as fully trained, while 644 (55.38%) required additional training and were classified as untrained. These 644 reports included clinical data that had not yet been fully processed or seen by the system. Thus, in the classification framework, the 238 true negatives refer to reports with no pathogen requiring interpretation, while the 644 are untrained clinical reports requiring interpretation but are not yet trained in the system (Table 7).

Performance Metrics:

- Precision: TP/(TP + FP) = 519/(519 + 0) = 1.0 (100%)
- Recall: TP/(TP + FN) = 519/(519 + 0) = 1.0 (100%)

These results indicate that the system achieved perfect accuracy in distinguishing between fully trained and untrained datasets under real-world conditions.

### 3.3. Element 3. Evaluation of the HITL Component in the Accuracy of Clinical Recommendations

Among the 1401 results analyzed, 238 (16.98%) were confirmed as negative and 1163 (83.02%) as positive. Of the positive results, 519 (44.62%) corresponded to fully trained data (Table 8. Additionally, 233 (20.03%) had been trained once but required further reinforcement. Specifically, 164 cases (14.10%) required one additional training session, 61 (5.24%) required two sessions, 7 (0.60%) required three, and 1 case (0.09%) required four sessions. A total of 267 cases (22.95%) were classified as partially untrained, defined as datasets with greater than 90% similarity to data previously seen by the system. Among these, 186 (15.99%) required two training sessions, 41 (3.52%) required three, 19 (1.63%) required four, 6 cases each (0.43%) required five or six sessions, respectively, and 11 (0.94%) required five training sessions. In contrast, 97 cases (8.34%) were classified as completely untrained, having less than 90% similarity to any known data set. These required more intensive training: 63 cases (5.42%) required two sessions, 15 (1.29%) required three, 12 (1.03%) required four, 4 (0.34%) required five, 2 (0.17%) required six, and 1 case (0.09%) required seven sessions. (Table 8). Additionally, 47 had untrained individual variables. Once trained, they were redistributed to one of the preceding categories.

Of the 644 reports that required training, all were reviewed by a clinical team with expertise in infectious disease. According to the consensus of the reviewers, no major discrepancies were identified. Minor discrepancies were observed in 100 (15.53%) of the 644 reports. Specifically, 11 (1.71%) reports involved the system recommending a different antibiotic than what was typically preferred; 35 (5.44%) reports suggested that an alternative antibiotic or combination could have been considered; 34 (5.28%) reports included recommendations where the dose or administration interval lacked formal FDA approval or varied across clinical references; and 20 (3.11%) reports did not address organisms of questionable pathogenicity (Table 9). Overall, these findings indicate a high level of consistency between the system’s recommendations and accepted clinical standards, with only minor variations that reflect the complexity and nuance of clinical decision-making.

## 4. Discussion

This study provides a robust assessment of the internal validation of this novel ML system, demonstrating its ability to accurately differentiate between trained and untrained data, both at the level of individual data points and in complex data sets. To our knowledge, this is the only system currently deploying such validation techniques.

It is important to note that once a data point is trained on the system, it remains trained even if it appears in larger data sets. This eliminates the need to retrain the system on the same data point in subsequent data sets. Furthermore, unlike traditional K-fold cross-validation, where training occurs on all but one fold, in this study, the data was trained on a single fold and tested against the other untrained folds. This approach was necessary due to the large volume of data and the nature of the system: once a data point is trained, it cannot be untrained. This method was more efficient and effective in terms of time, allowing for more training sessions within the constraints of the system.

The system shows high accuracy in recognizing new data by distinguishing untrained from trained individual data points using multiple validation techniques (K-fold cross-validation, random subsampling, and holdout validation). It also accurately classifies complex data sets, with over 1000 real-world laboratory data points from diverse sources, which highlights its ability to generalize and avoid overfitting to the training data. Also, the diversity of real-world data with data from 66 laboratories in 55 regions, across multiple states and one international location, ensures the system’s adaptability to diverse clinical settings and reduces the possibility of bias [19].

The use of multiple validation techniques (K-fold cross-validation, random subsampling, and holdout validation) in this study mitigates the risks of overfitting and improves the generalizability of the model [7,20]. In the system studied, the HITL component for multi-stage data training enables the participation of clinical experts, prevents error propagation, and ensures compliance with current medical guidelines [4]. This is a key strength, particularly in the context of high-stakes clinical decision-making [21,22].

The critical importance of HITL validation is highlighted by the detection of 100 minor discrepancies out of 644 reviewed reports. This finding reinforces that, although ML algorithms are powerful tools capable of processing large volumes of data and identifying patterns, the nuanced clinical judgment of infectious disease specialists remains essential to ensure patient safety and optimize treatment strategies [3,23]. The nature of these discrepancies is particularly telling: while the automated system excels at data analysis, it lacks the ability to account for complex clinical subtleties and evolving scientific insights. For instance, the system relies on external references, such as FDA guidelines or the published literature, for dosing and administration intervals. Although generally accurate, these references may not always reflect the most current evidence or account for patient-specific factors, which clinicians are uniquely positioned to evaluate. Additionally, the system provides generalized recommendations and is not designed to handle rare or atypical clinical scenarios, further emphasizing the necessity of expert oversight. These results suggest that the HITL process successfully mitigated potential errors or suboptimal recommendations, underscoring the vital role of human oversight in AI-supported clinical care [22,24]. This is especially significant in the realm of antimicrobial stewardship, where inappropriate antibiotic prescribing can contribute to the growing threat of antimicrobial resistance [22,25].

The methodology and findings of this study can be contrasted with previous attempts to validate ML-based CDSSs; for example, IBM Watson for Oncology, while initially promising, failed due to inaccurate recommendations and integration challenges, ultimately leading to its discontinuation [26,27]. This highlights the crucial need for robust validation processes, as demonstrated in the present study. The Mayo Clinic Predictive Analytics project faced data complexity and integration issues, which halted its progress [23]. The Pediatric Alert System employed cross-validation techniques; however, this system suffered from limitations due to data sparsity, which impacted the effectiveness of its model [28]. The use of large, diverse datasets and HITL helps overcome this.

This study demonstrates a robust validation process, which emphasizes real-time application and human oversight. It also highlights the advantage of having a system that is not intended to be used for the “discovery” of new findings but instead focuses on adhering to existing guidelines and best practices. The system was carefully trained by infectious disease experts and periodically re-evaluated. This method ensures both the accuracy and up-to-date nature of the database and algorithms. The system was designed to assume that error is possible and, therefore, never allows data to be trained indefinitely.

Limitations of this study include that data curation, particularly for complex data sets, was restricted to a single-day period. Although the sample was geographically diverse, limiting the sample to a single day could introduce some bias into the data sample, possibly related to the capabilities of the quality control team on that particular day. Furthermore, while HITL is essential, the manual review process introduces the potential for bias and variability in data interpretation [7,29]. Automating some components of the HITL process could improve consistency. Also, despite the perfect performance metrics reported, the possibility of data leakage or oversimplification (e.g., binary classification as “seen” vs. “new”) cannot be excluded. This may lead to overfitting and limit generalizability, highlighting the need for future external validation. Furthermore, the model’s architecture, hyperparameter settings, and handling of class imbalance were not disclosed in full due to proprietary constraints. Lastly, this study did not assess downstream clinical outcomes—such as mortality, length of hospital stay, or antibiotic consumption—which are essential to evaluate the real-world effectiveness and impact of the CDSS. Future prospective studies will be necessary to address this limitation.

The system’s unique approach to antimicrobial guidance is an innovative system, notable for its reliance on established treatment guidelines and avoidance of novel treatment suggestions, thereby prioritizing safety and adherence to proven practices. To meet these goals, this first study demonstrates that the first two steps of the process work robustly, laying the groundwork to demonstrate that the system works efficiently, and if subsequent steps follow suit, this will lead to appropriate and useful recommendations in the real world. This is a preliminary study that focuses primarily on the technical performance of the system in identifying trained versus untrained data, and final results and clinical outcomes are not yet included. Therefore, the impact of the system on patient care and antimicrobial stewardship requires further study.

This study provides valuable insights into the design and validation of ML-based CDSSs for antimicrobial stewardship. The combination of robust statistical validation techniques with a comprehensive HITL process represents a promising strategy for ensuring the accuracy and clinical relevance of AI-driven recommendations. The findings suggest that such systems can potentially contribute to improved antimicrobial prescribing practices and, ultimately, better patient outcomes.

Future research should focus on external validation and evaluating the performance of the model in diverse clinical settings and patient populations to assess its generalizability and identify potential biases [20,30,31]. In addition, prospective clinical trials through randomized controlled trials to evaluate the impact of the CDSS on antimicrobial use, patient outcomes, and healthcare costs may be of benefit [4,22,32]. Lastly, expanding HITL evaluation by collecting more detailed data on the types of discrepancies identified during the HITL process can further refine the model and identify areas where clinician training may be needed. Integration with Electronic Health Records (EHRs) may facilitate seamless data flow and improve the clinician workflow [26,33,34,35].

By continuing to refine and validate these systems, we can harness the power of AI to support clinicians in making informed decisions about antimicrobial therapy, contributing to the fight against antimicrobial resistance and improving patient care.

## 5. Conclusions

This study demonstrates the robust performance of a novel ML model in accurately distinguishing between trained and new data, achieving 100% accuracy. This result was validated using various methodologies, including K-fold cross-validation, random subsampling, and holdout validation. The system also successfully identified complex data sets as trained or untrained with high reliability. The integration of HITL validation improved model adaptability and quality control, reinforcing the value of clinical oversight in AI systems. However, HITL can also introduce some variability in data interpretation. Therefore, ongoing review is important to ensure alignment with the most up-to-date clinical practice recommendations and to optimize HITL interventions. These findings suggest that advanced ML models can be trained effectively and consistently to support clinical decision support (CDS) systems. Although the system demonstrated excellent performance, with 100% accuracy in avoiding major discrepancies and 84% agreement for minor discrepancies with clinical interpretation, future research should focus on its external validity, as this tool only interprets, to the best of its ability, the complex molecular data on bacteria and their resistance genes. Further studies should focus on its use in routine clinical practice and its utility as a tool for antimicrobial stewardship.

## Figures and Tables

**Table 1:** FDA-approved BioFire panels.

Panel	Sample Type	Number of Targets	Key Individual Targets
Respiratory 2.1 (RP2.1)	Nasopharyngeal swab	22	The respiratory pathogens included Adenovirus (AdV); seasonal Coronaviruses (229E, HKU1, NL63, and OC43); Severe Acute Respiratory Syndrome Coronavirus 2 (SARS-CoV-2); Human Metapneumovirus (hMPV); Human Rhinovirus/Enterovirus (HRV/EV); Influenza A Virus (Flu A) with subtypes A/H1, A/H3, and A/H1-2009; Influenza B Virus (Flu B); Parainfluenza Virus (PIV) types 1, 2, 3, and 4; and Respiratory Syncytial Virus (RSV). The bacterial targets were *Bordetella Parapertussis*, *Bordetella Pertussis*, *Chlamydia Pneumoniae* (*C. pneumoniae*), and *Mycoplasma Pneumoniae* (*M. pneumoniae*).
Blood Culture (BCID2)	Positive blood culture	43	The Gram-negative bacteria included the following: *Acinetobacter calcoaceticus–baumannii complex*, *Bacteroides fragilis*, Enterobacterales, *Enterobacter cloacae complex*, *Escherichia coli* (*E. coli*), *Klebsiella aerogenes*, *Klebsiella oxytoca*, *Klebsiella pneumoniae* group, *Proteus* spp., *Salmonella* spp., *Serratia marcescens*, *Haemophilus influenzae*, *Neisseria meningitidis*, *Pseudomonas aeruginosa* (*P. aeruginosa*), and *Stenotrophomonas maltophilia*. The Gram-positive bacteria included the following: *Enterococcus faecalis*, *Enterococcus faecium*, *Listeria monocytogenes*, *Staphylococcus* spp., including *Staphylococcus aureus* (*S. aureus*), *Staphylococcus epidermidis*, and *Staphylococcus lugdunensis*; and *Streptococcus* spp., including *Streptococcus agalactiae*, *Streptococcus pneumoniae* (*S. pneumoniae*), and *Streptococcus pyogenes*. Yeasts included the following: *Candida albicans*, *Candida auris*, *Candida glabrata*, *Candida krusei*, *Candida parapsilosis*, *Candida tropicalis*, and *Cryptococcus* spp. (*C. neoformans*/*C. gattii*). Resistance genes detected included the following: carbapenemases (*IMP*, *KPC*, *OXA-48*-like, *NDM*, *VIM*), colistin resistance (*mcr-1*), extended-spectrum beta-lactamases (ESBLs), such as *CTX*-*M*, methicillin resistance (*mecA/C* and MREJ for MRSA), and vancomycin resistance (*vanA/B*).
Gastrointestinal (GI)	Stool in Cary–Blair medium	22	The bacterial pathogens included the following: *Campylobacter* spp. (*C. jejuni*, *C. coli*), Clostridioides (*Clostridium difficile* (toxin A/B), Plesiomonas shigelloides, *Salmonella* spp., *Vibrio* spp. (*V. parahaemolyticus*, *V. vulnificus*, *V. cholerae*), *Vibrio cholerae*, *Yersinia enterocolitica*, and diarrheagenic *Escherichia coli*/*Shigella pathotypes*: Enteroaggregative *E. coli* (EAEC), *Enteropathogenic E. coli* (EPEC), *Enterotoxigenic E. coli* (ETEC; lt/st), Shiga toxin–producing *E. coli* (STEC; *stx1*/*stx2*), *E. coli* O157, and Shigella/Enteroinvasive *E. coli* (EIEC). The viral targets included the following: Adenovirus F40/41, Astrovirus, Norovirus GI/GII, Rotavirus A, and Sapovirus (genogroups I, II, IV, and V). Parasitic pathogens included the following: *Cryptosporidium* spp., *Cyclospora cayetanensis*, *Entamoeba histolytica*, and *Giardia lamblia*.
Meningitis/Encephalitis (ME)	CSF	21	The bacterial pathogens included the following: *Escherichia coli* K1, *Haemophilus influenzae*, *Listeria monocytogenes*, *Neisseria meningitidis*, *Streptococcus agalactiae*, and *Streptococcus pneumoniae*. The viral targets were Cytomegalovirus (CMV), Enterovirus (EV), Herpes Simplex Virus type 1 (HSV-1), Herpes Simplex Virus type 2 (HSV-2), Human Herpesvirus 6 (HHV-6), Human Parechovirus (HPeV), and Varicella-Zoster Virus (VZV). The yeast panel included the following: *Cryptococcus* spp. (*C. neoformans*/*C. gattii*).
Pneumonia (PN)	BAL/sputum	33	The semi-quantitative bacterial pathogens included the following: Acinetobacter calcoaceticus–baumannii complex, Enterobacter cloacae complex, *Escherichia coli* (*E. coli*), *Haemophilus influenzae*, *Klebsiella aerogenes*, *Klebsiella oxytoca*, *Klebsiella pneumoniae* group, *Moraxella catarrhalis*, *Proteus* spp., *Pseudomonas aeruginosa* (*P. aeruginosa*), *Serratia marcescens*, *Staphylococcus aureus* (*S. aureus*), *Streptococcus agalactiae*, *Streptococcus pneumoniae* (*S. pneumoniae*), and *Streptococcus pyogenes*. The qualitative atypical bacteria included the following: *Chlamydia pneumoniae*, *Legionella pneumophila*, and *Mycoplasma pneumoniae*. Viruses included the following: Adenovirus, Coronavirus, Human metapneumovirus (hMPV), Human rhinovirus/enterovirus (HRV/EV), Influenza A virus (Flu A), Influenza B virus (Flu B), Parainfluenza virus (PIV), and Respiratory syncytial virus (RSV). Antimicrobial resistance genes detected included the following: carbapenemases (*IMP*, *KPC*, *NDM*, *OXA-48*-like, *VIM*), extended-spectrum beta-lactamase (ESBL) genes such as *CTX-M*, and methicillin resistance markers *mecA/C* and MREJ for methicillin-resistant *Staphylococcus aureus* (MRSA).
Joint infection (JI)	Sinovial liquid	39	Gram-positive bacteria included the following: *Anaerococcus prevotii*/*vaginalis*, *Clostridium perfringens*, *Cutibacterium avidum*/*granulosum*, *Enterococcus faecalis*, *Enterococcus faecium*, *Finegoldia magna*, *Parvimonas micra*, *Peptoniphilus* spp., *Peptostreptococcus anaerobius*, *Staphylococcus aureus*, *Staphylococcus lugdunensis*, *Streptococcus* spp., *Streptococcus agalactiae*, *Streptococcus pneumoniae*, and *Streptococcus pyogenes*. Gram-negative bacteria included the following: *Bacteroides fragilis*, *Citrobacter* spp., Enterobacter cloacae complex, *Escherichia coli*, *Haemophilus influenzae*, *Kingella kingae*, *Klebsiella aerogenes*, *Klebsiella pneumoniae* group, *Morganella morganii*, *Neisseria gonorrhoeae*, *Proteus* spp., *Pseudomonas aeruginosa*, *Salmonella* spp., and *Serratia marcescens*. Yeasts included the following: *Candida* spp., particularly *Candida albicans*. Detected resistance genes included the following: carbapenemases (*KPC*, *NDM*, *IMP*, *OXA-48*-like, *VIM*), extended-spectrum beta-lactamases (ESBL, *CTX-M*), methicillin resistance (*mecA/C* and MREJ, indicative of MRSA), and vancomycin resistance (*vanA/B*).

**Table 2:** Summary of processes in element 1.

**N data points from BioFire’s six different panels** **N unique data points noted after redundancies across the panels** Brackets were placed around data points to ensure no overfitting, and they are new to the system.

Training Session 1: All unique data points were entered as a single data set. System performance: The system identified all data as new. Training Session 2: The data points were divided into BioFire’s corresponding diagnostic panels (respiratory, blood, CNS, joint, etc.). System performance: The system identified all data as new within their respective panels.

The data set was then split into randomized groups, referred to as K-folds, for cross-validation. Training session 3: K-fold 1 was used for training and then tested against the data in the remaining untrained K-folds.

System performance: Only the data from K-fold 1 was recognized as trained; all other data remained untrained.
Training sessions 4–7: The process described in session 3 was repeated independently for K-folds 2, 3, 4, and 5. System performance: In each case, the system identified only the data from the trained K-fold as trained. All remaining K-folds were unrecognized (i.e., untrained). Training session 8: Random untrained data points were introduced into the previously trained K-folds and tested. System performance: The system recognized only the previously trained data, while the newly introduced data was initially untrained and subsequently learned. Training session 9: All data were reintroduced into the system collectively as a single data set. System performance: The system recognized all data points as previously trained.

**Table 3:** Randomized data set, grouped into sets.

Data Set	# Variables	Description of Variables
K fold 1	21	*Staphylococcus aureus*, *Clostridium perfringens*, *Cryptosporidium*, Varicella zoster virus (VZV), *Cryptococcus* (*C. neoformans*/*C. gattii*), *Shigella*/*Enteroinvasive E. coli* (*EIEC*), *Neisseria gonorrhoeae*, *Vibrio* (*V. parahaemolyticus*/*V. vulnificus*/*V. cholerae*), Human metapneumovirus, *Klebsiella oxytoca*, *Enterococcus faecalis*, Parainfluenza virus 1, *Candida parapsilosis*, *Klebsiella aerogenes*, *Enterobacter cloacae complex*, *Haemophilus influenzae*, Adenovirus F40/41, Coronavirus 229E, *IMP*, *mcr*-*1.*
K fold 2	19	Influenza A virus A/H3, *Clostridioides* (*Clostridium*) *difficile* (*toxin A*/*B*), *Streptococcus agalactiae*, Adenovirus, *Bordetella pertussis*, *Candida krusei*, Herpes simplex virus 2 (HSV-2), *Serratia marcescens*, Cytomegalovirus (CMV), *Parainfluenza virus 2*, *Moraxella catarrhalis*, *Staphylococcus lugdunensis*, Human herpesvirus 6 (HHV-6), *Bacteroides fragilis*, *Campylobacter* (*C. jejuni*/*C. coli*/*C. upsaliensis*), *Candida albicans*, *Enteroaggregative E. coli* (*EAEC*), *Coronavirus OC43*, *OXA*-*48*-like
K fold 3	18	Human rhinovirus/enterovirus, *Vibrio cholerae*, *Mycoplasma pneumoniae*, Influenza B virus, *Legionella pneumophila*, *Chlamydia pneumoniae*, *Candida tropicalis*, *KPC*, *Plesiomonas shigelloides*, *Shiga*-like toxin-producing *E. coli* (STEC) *stx1*/*stx2*, *Enteropathogenic E. coli* (EPEC), *Cyclospora cayetanensis*, *Enterobacterales*, *Anaerococcus prevotii*/*vaginalis*, *Cutibacterium avidum*/*granulosum*, Parainfluenza virus 3, *VIM*, *NDM*
k-fold 4	12	*Streptococcus pyogenes*, *Enterococcus faecium*, Influenza A virus A/H1, Rotavirus A, *Staphylococcus epidermidis*, Human parechovirus (HPeV), *Klebsiella pneumoniae group*, *Neisseria meningitidis*, *Candida auris*, *Bordetella parapertussis*, *Peptostreptococcus anaerobius*, Coronavirus NL63.
K fold 5	14	Norovirus GI/GII, *Candida glabrata*, *Escherichia coli*, *Peptoniphilus*, *Acinetobacter calcoaceticus–baumannii complex*, *Streptococcus* spp., *Pseudomonas aeruginosa*, *Escherichia coli K1*, Herpes simplex virus 1 (HSV-1), *E. coli* O157, Parainfluenza virus 4, *Streptococcus pneumoniae*, Coronavirus HKU1, Severe Acute Respiratory Syndrome Coronavirus 2 (SARS-CoV-2).
Holdout	27	Influenza A virus A/H1-2009, Influenza A virus, *Proteus* spp., *Stenotrophomonas maltophilia*, Respiratory syncytial virus, *Listeria monocytogenes*, *Staphylococcus* spp., Astrovirus, Sapovirus (I, II, IV, and V), *Enterotoxigenic E. coli* (*ETEC*) *lt*/*st*, *Entamoeba histolytica*, *Giardia lamblia*, *Yersinia enterocolitica*, Enterovirus (EV), Coronavirus, *Citrobacter*, *Kingella kingae*, *Morganella morganii*, *Candida* spp., *Finegoldia magna*, *Parvimonas micra*, *CTX*-*M*, *mecA*/*C*, *vanA*/*B*, ESBL, *Klebsiella pneumonia group*, *Salmonella.*

**Table 4:** Definition of status.

	Auto-Approve Fully Trained Data Points and Data Sets	Auto-Match Partially Trained Data Sets with Completed Trained Data Points	High Confidence Untrained Data Sets with Trained Data Points	New Untrained Data Sets and Untrained Data Points
Required training session	Completed full training of all data points and data sets (at least two data set training sessions)	One data set training session was completed; however, at least one more training session is required	≥90 percent like previously trained data sets where data points are trained completely	Data sets and points require full training

**Table 5:** Proportion of true positive results to total expected positives.

Folds	True Positives (Identified Trained Data)	True Negative (Identified New Data)	False Positive (Identified New Data as Trained Data)	False Negatives (Identified Trained Data as New Data)
Fold 1	21	21	0	0
Fold 2	19	19	0	0
Fold 3	18	18	0	0
Fold 4	12	12	0	0
Fold 5	14	14	0	0
Holdout	27	27	0	0
Total	111	111	0	0

TP: true positives, TN: true negatives, FP: false positives, FN: false negatives.

**Table 6:** Performance metric based on confusion matrix results.

Metric	Formula	Result
Precision	TP/TP + FP	111/(111 + 0) = 1.00 (100%)
Recall (Sensitivity)	TP/TP + FN	111/(111 + 0) = 1.00 (100%)
F1 Score	2 × Precision × Recall/ Precision + Recall)	2 × 1 × 1/(1 + 1) = 1.00 (100%)
Positive Predictive Value	TP/(TP + FP)	111/(111 + 0) = 1.00 (100%)
Negative Predictive Value	TN/(TN + FN)	111/(111 + 0) = 1.00 (100%)

TP: true positives, TN: true negatives, FP: false positives, FN: false negatives.

**Table 7:** Classification performance of the system on 1401 diagnostic reports.

Classification Outcome	Count	Description
True Positives (TP)	519	Fully trained reports correctly identified as trained
True Negatives (TN)	238	Negative reports without relevant pathogens
False Positives (FP)	0	No untrained reports were incorrectly identified as trained
False Negatives (FN)	0	No trained reports were misclassified as untrained
Untrained (correctly flagged)	644	Reports requiring training correctly identified as untrained
Total reports	1401	

**Table 8:** Number of steps with human intervention required before fully trained.

			Number of Trainings						
Variable	n	%	1	2	3	4	5	6	7
Total	1401	100.00							
Negative	238	16.98							
Positive	1163	83.02							
Complete trained data	519	44.63							
Trained once but required additional training	233	20.03	164	61	7	1			
Partially untrained data	267	22.95	186	41	19	5	11	5	
Completely untrained data	97	8.34		63	15	12	4	2	1

**Table 9:** Frequency of major discrepancies and minor discrepancies.

Discrepancy	Frequency	%
Major discrepancy		
No discrepancy	644	100.00
A known pathogen has NOT been addressed	0	0
The recommended antibiotic has NO activity against the microbe detected	0	0
Minor discrepancy		
No discrepancy	544	84.47
An alternative to OneChoice could have been recommended	11	1.71
Among the alternative recommendations, another antibiotic or a combination of antibiotics could have been recommended	35	5.44
Dosing and length of therapy are not consistent with the FDA guidelines or other literature	34	5.28
Microbes that should have been targeted were NOT addressed	20	3.11
Total	100	15.53

## Data Availability

All data used in this study were either publicly available (BioFire panels) or obtained from internal, de-identified laboratory submissions. No protected health information (PHI) was accessed or disclosed. The data analyzed in this manuscript, as well as its definitions, can be downloaded at the following link: Supplement S2.

## Supplementary Materials

The following supporting information can be downloaded at https://www.mdpi.com/article/10.3390/life15071123/s1, Supplement S1, Supplement S2, Supplement S3.

## Abbreviations

ML	Machine learning
CDSS	Clinical decision support system
HITL	Human involvement in the loop
AI	Artificial intelligence
ASP	Antimicrobial stewardship program
AMR	Antimicrobial resistance
