# Supplementary material for: Internal Validation of a Machine Learning-Based CDSS for Antimicrobial Stewardship
Source: Life (Basel). 2025 Jul 17;15(7):1123. doi: 10.3390/life15071123 (PMC12298242; doi:10.3390/life15071123)
Supplement: Supplementary file 1 [file life-15-01123-s001.zip › Supllemnt 3 Validation study/Tables that need to be included.pdf]

Table S1. Recall of trained vs untrained data points

Session#	Type	New data	Recall trained data	Recall untrained data
1	All data	111	0	111
2	Panels	111	0	111
3	Fold 1	21	21	90
4	Fold 2	19	40	71
5	Fold 3	18	58	53
6	Fold 4	12	70	41
7	Fold 5	14	84	27
8	Hold-out	27	111	0
9	All data	0	111	0
	Total	333	495	282

Table S2. Proportion of true positive results to total expected positives

	True positives (identified trained data)	True negative (Identified new data)	False positive (Identified new data as trained data)	False negatives (Identified trained data as new data)
Fold 1	21	21	0	0
Fold 2	19	19	0	0
Fold 3	18	18	0	0
Fold 4	12	12	0	0
Fold 5	14	14	0	0
Hold-out	27	27	0	0
Total	111	111	0	0

https://doi.org/10.3390/xxxxx
