# Supplementary material for: Internal Validation of a Machine Learning-Based CDSS for Antimicrobial Stewardship
Source: Life (Basel). 2025 Jul 17;15(7):1123. doi: 10.3390/life15071123 (PMC12298242; doi:10.3390/life15071123)
Supplement: Supplementary file 1 [file life-15-01123-s001.zip › Supllemnt 3 Validation study/Validation Document Overview_ Element One.pdf]

Validation Document Overview: Element One

This document serves as an explainer for the validation screenshots related to **Element One** of the study. It outlines how novel data was introduced, identified, and iteratively trained within the system using a K-fold methodology. The intent of the validation is to ensure the system accurately recognizes untrained input and appropriately updates its status as training progresses.

Data Import and Novelty Detection

To validate Element One, six distinct data sets were imported into the system. These data sets were intentionally structured to resemble **novel, untrained inputs**, ensuring the model had no prior exposure to them. This was done by placing brackets around the data points. This isolation was critical to assessing whether the system could accurately distinguish unfamiliar content.

Upon import, the system successfully identified all entries, including both **organisms** and **resistance genes**, as new data. This status was marked by a **star icon (☆)** next to each data point. The presence of the star informs the Quality Assurance (QA) reviewer that these entries are unrecognized by the model and require training. The fact that all six datasets triggered the "novel data" flag confirms the system's effective separation of training and validation environments.

Symbol Legend and Training Status Indicators

The system uses intuitive visual indicators to show the status of each data point throughout the training process:

- **Star (☆)**: Represents untrained, novel data. The model does not currently recognize or respond to this input.
- **Triangle (▲)** or **"I" symbol**: Denotes that the data point has been successfully trained and is now recognized by the system.

This transition from "untrained" to "trained" status provides a transparent and traceable way to monitor learning progress and system responsiveness.

K-Fold Training Framework

The training process is structured using a **K-fold validation method**, in which each fold is represented by a separate folder. Within each fold:

- Selected data points from the six datasets are trained and re-entered into the system.

- Once trained, the star symbol is replaced by the triangle or “I” icon, confirming that the system has learned the input.

This process is repeated until every data point has been trained and accurately recognized across all K folds. This technique ensures rigorous validation by training on subsets while evaluating on the remaining unseen data.

System Precision and Recall Integrity

As demonstrated by the validation documents, the system exhibits **exceptional precision and recall performance**, achieving **100% accuracy** in identifying, training, and recalling data. This level of precision is both expected and reliable in modern technology systems.

To draw a comparison: much like your email account accurately suggests addresses based on your typing, or how your computer knows with absolute certainty which key you've pressed, this system is designed with similar logic. It was built to provide deterministic responses to well-defined inputs, resulting in robust, repeatable accuracy. The elementary yet powerful architecture underlying this functionality ensures that once data is trained, the system responds with **perfect reliability**.

Proprietary Considerations

Due to the **proprietary nature of Arkstone’s technology**, the underlying codebase cannot be publicly shared. However, the attached validation documents themselves serve as **direct evidence** of the system’s accuracy and operational soundness. Each screenshot captures the system’s ability to recognize, learn, and recall novel data with unerring precision, reinforcing confidence in the architecture and methodology used.
