# Supplementary material for: Internal Validation of a Machine Learning-Based CDSS for Antimicrobial Stewardship
Source: Life (Basel). 2025 Jul 17;15(7):1123. doi: 10.3390/life15071123 (PMC12298242; doi:10.3390/life15071123)
Supplement: Supplementary file 1 [file life-15-01123-s001.zip › Supplement 1 Sample OneChoice Report.pdf]

ARKSTONE

Antimicrobial Stewardship: Molecular Lab Result

**Report ID**	XXXXXXX	**Patient**	XXXXX, XXXXX	**DOB**	XX/XX/XXXX	**Collected**	XX/XX/XXXX
**Specimen Type**	Urine	**Provider**	XXXXX, XXXXX	**Resulted**	XX/XX/XXXX	**Received**	XX/XX/XXXX

Infection Complexity **ARK**SCORE™

LO 5 HI

Organisms Detected

Common pathogens in bold

- Candida albicans
- **Escherichia coli**
- **Klebsiella pneumoniae**

Resistance Detected

Extended-Spectrum Beta-Lactamase

Ampicillin/Cephalosporin

Antimicrobial Resistance **ARK**SCORE™

LO 7 HI

Allergies Reported

Macrolide

OneChoice Drug Info

Trimethoprim-Sulfamethoxazole

Dose Adj ☑ Renal ☐ Hepatic

Interactions ACE inhibitors

Avg Price $ $ $ $

Adverse Reaction **ARK**SCORE™

LO 2 HI

ONECHOICE® PLUS

Dose adjustments, other drug options, references, translations and support

ONECHOICE®

Trimethoprim-Sulfamethoxazole DS 1 tab PO BID x 3 days for possible simple UTI

See additional dosing info on OneChoice Plus

Alternative Treatment Options with Adverse Reaction Arkscore™

- **Macrobid°** ARKSCORE 3 100 mg PO Q12H x 5 days for possible simple UTI
- **Fosfomycin°** ARKSCORE 1 3 gm PO x 1 dose for possible simple UTI
- **Cipro°** ARKSCORE 5 250-500 mg PO Q12H x 3 days for possible simple UTI
- **Gentamicin°** ARKSCORE 5 5-7 mg/kg/dose IV/IM Q24H (5 mg/kg/dose may be sufficient) x 1 dose for possible simple UTI
- **Ertapenem** ARKSCORE 3 1 gm IV/IM Q24H x 5-14 days for possible complicated UTI
- **Fluconazole°** ARKSCORE 1 200-400 mg PO daily x 14 days for possible candiduria (although common colonizer/contaminate)

° See additional dosing info on OneChoice Plus. Macrobid efficacy against Klebsiella pneumoniae and E. coli is uncertain. Fosfomycin efficacy against Klebsiella pneumoniae is uncertain. Gentamicin efficacy against E. coli is uncertain.

Why is this the OneChoice?

E. coli and Klebsiella pneumoniae can be pathogenic when found in urine samples. Candida was not targeted since it may be a colonizer, found naturally, or a possible contaminant. If concerned, treatment modifications may be needed. Resistance genes were detected in multiple classes which may limit available treatment options. The reported allergies do not preclude any treatment options. TMP-SMX efficacy against Klebsiella pneumoniae and E. coli is uncertain. ‡

When should this be treated?

Asymptomatic bacteriuria does not typically need treatment, and microbe detection may not indicate infection. However, treatment may be necessary during pregnancy or prior to urological procedures. Simple UTIs are typically treated for 3 days (fluoroquinolones/TMP-SMX), or 5 days (beta-lactams). In more complicated cases therapy may be extended to 7-14 days. ‡

Are there any special considerations?

Multiple microbes detected may indicate contamination or colonization. As ESBL resistance is on the CDC threat list, tracking and monitoring may be indicated if possible. Amp/ceph and ESBL can be associated with other resistance genes. Antibiotics should therefore be used with caution as drug failure is possible. Resistance detected may only affect certain microbes, and in some cases, none at all. ‡

Infection Control Precautions: ☑ Standard ☑ Contact

* Dosing and duration of treatment based on adult patient, with no medical history, normal BMI, renal and hepatic functions, and minimal time required to treat simple infections. Treatment is directed at common pathogens noted above, and the most commonly associated antibiotic resistance based on genes detected. Resistance is variable and drug failure is possible. Additional microbiology workup and treatment modification may be needed. Visit OneChoice Plus for expanded information.

‡ For education purposes only. This is not a diagnosis. Clinical correlation and physician judgment required when making diagnosis or treatment decisions. Recommendations based on lab results, and limited to specimen source, organisms, resistance, allergies, and ICD10 codes. Patient has not been examined nor their medical history reviewed.

Copyright 2025 Arkstone Medical Solutions. OneChoice, MedsMatrix, and ArkScore are based on patent pending methods and algorithms. Learn more at arkstone.ai/report
