# Supplementary figures and images for: Internal Validation of a Machine Learning-Based CDSS for Antimicrobial Stewardship
Source: Life (Basel). 2025 Jul 17;15(7):1123. doi: 10.3390/life15071123 (PMC12298242; doi:10.3390/life15071123)

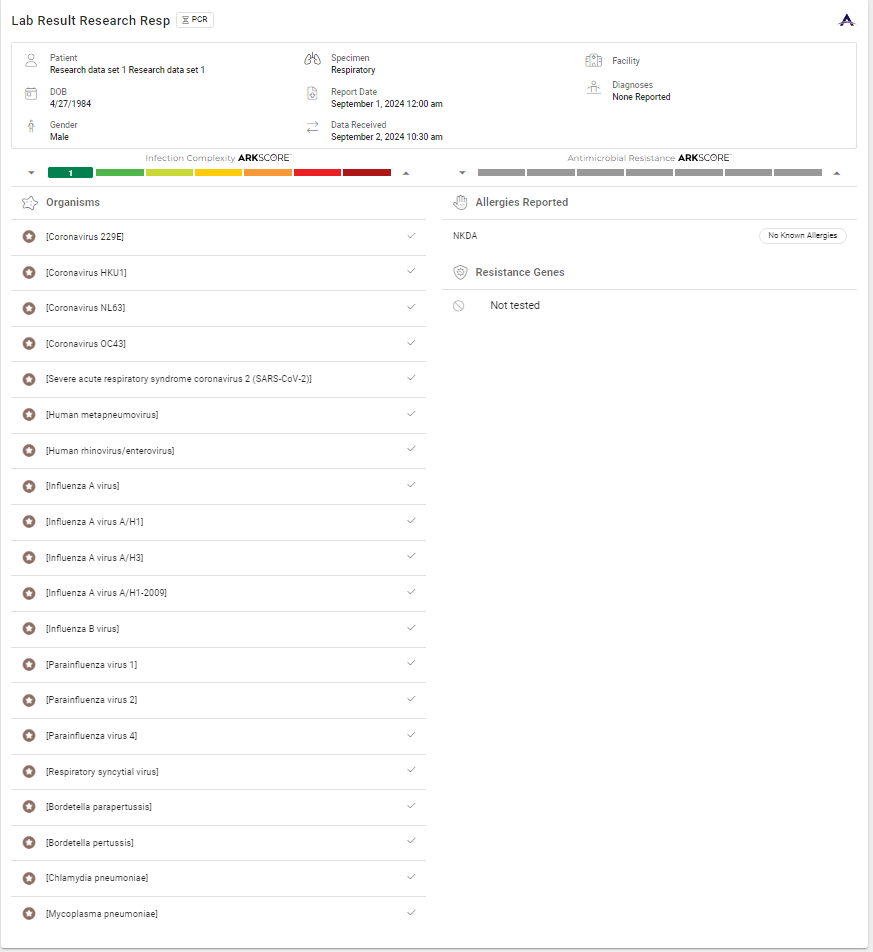

Supplement: Supplementary file 1 [file life-15-01123-s001.zip › Supllemnt 3 Validation study/Data set 1.PNG]

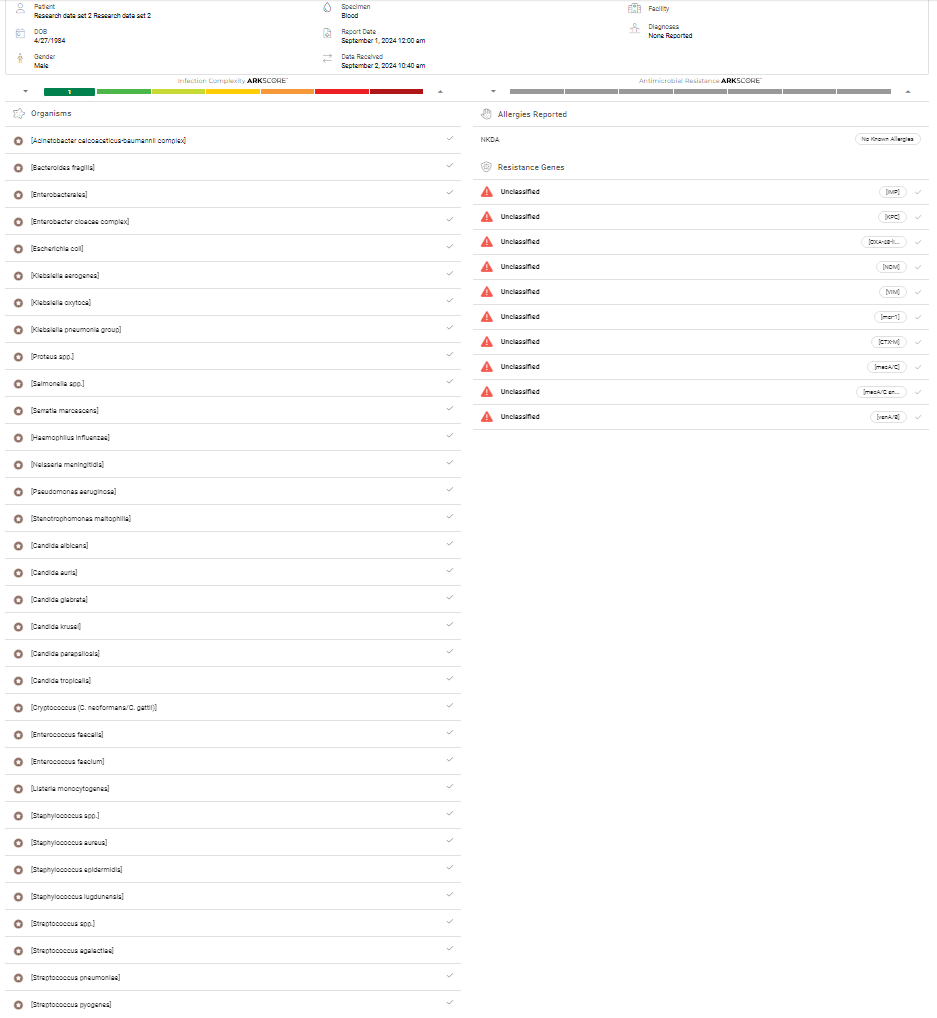

Supplement: Supplementary file 1 [file life-15-01123-s001.zip › Supllemnt 3 Validation study/Data set 2.PNG]

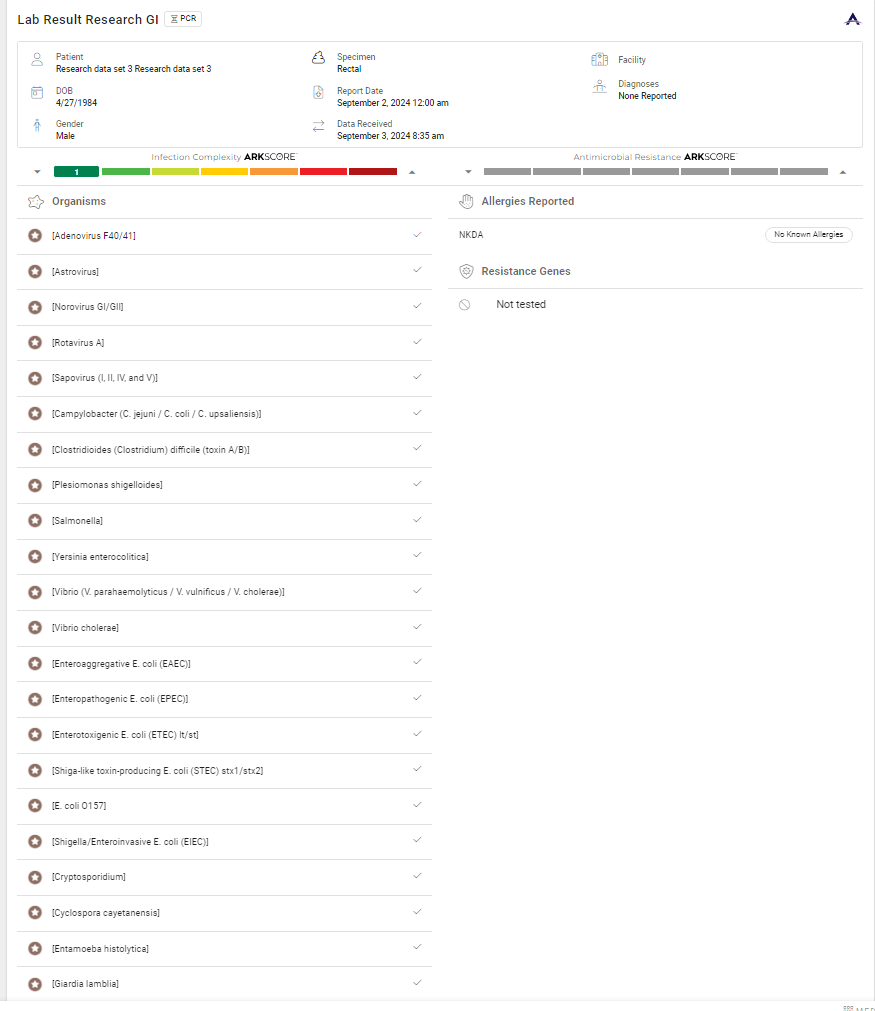

Supplement: Supplementary file 1 [file life-15-01123-s001.zip › Supllemnt 3 Validation study/Data set 3.PNG]

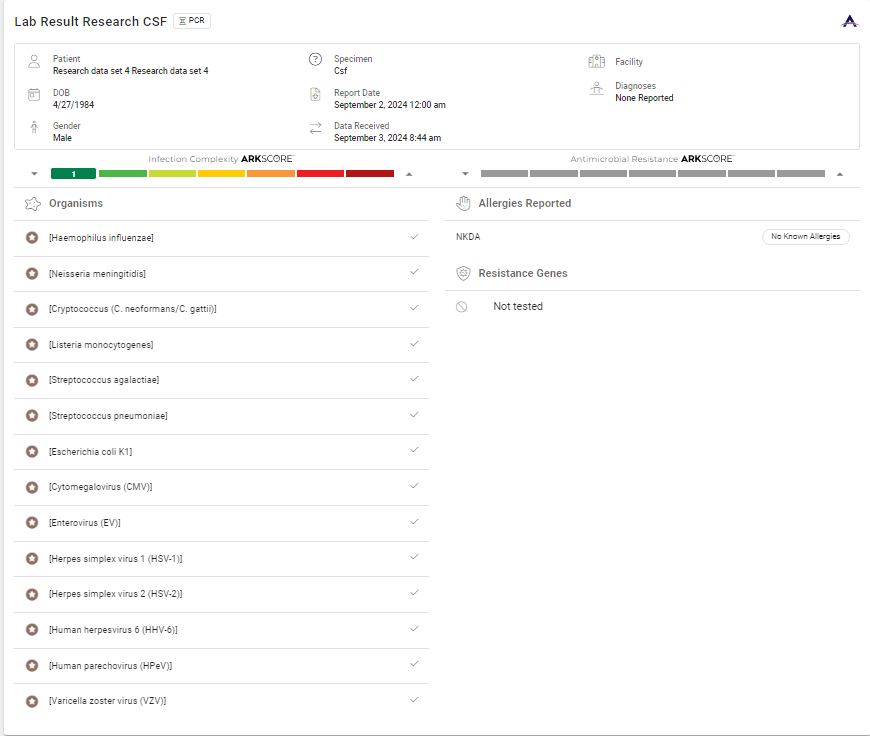

Supplement: Supplementary file 1 [file life-15-01123-s001.zip › Supllemnt 3 Validation study/Data set 4.PNG]

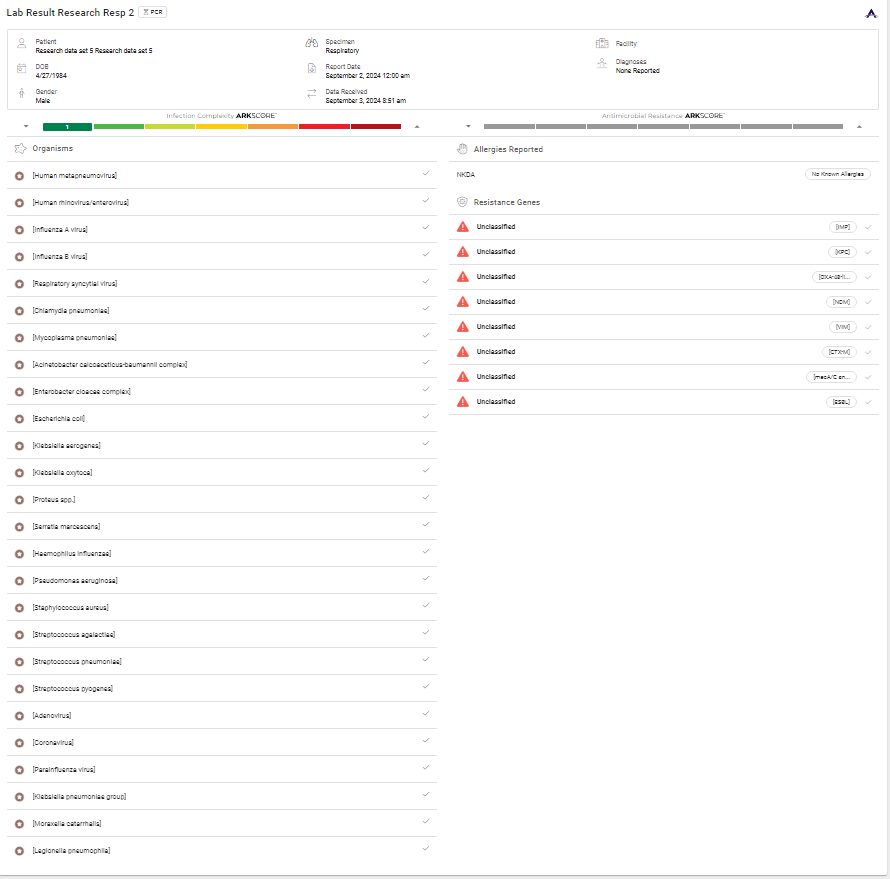

Supplement: Supplementary file 1 [file life-15-01123-s001.zip › Supllemnt 3 Validation study/Data set 5.PNG]

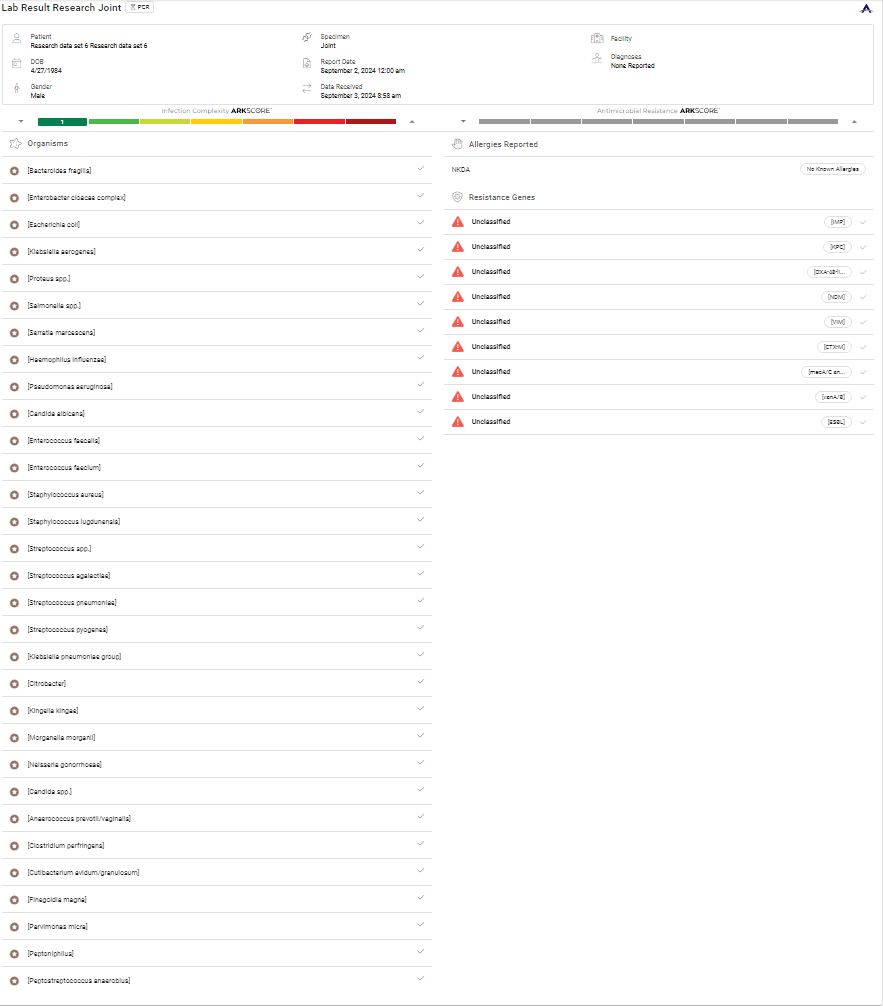

Supplement: Supplementary file 1 [file life-15-01123-s001.zip › Supllemnt 3 Validation study/data set 6.PNG]

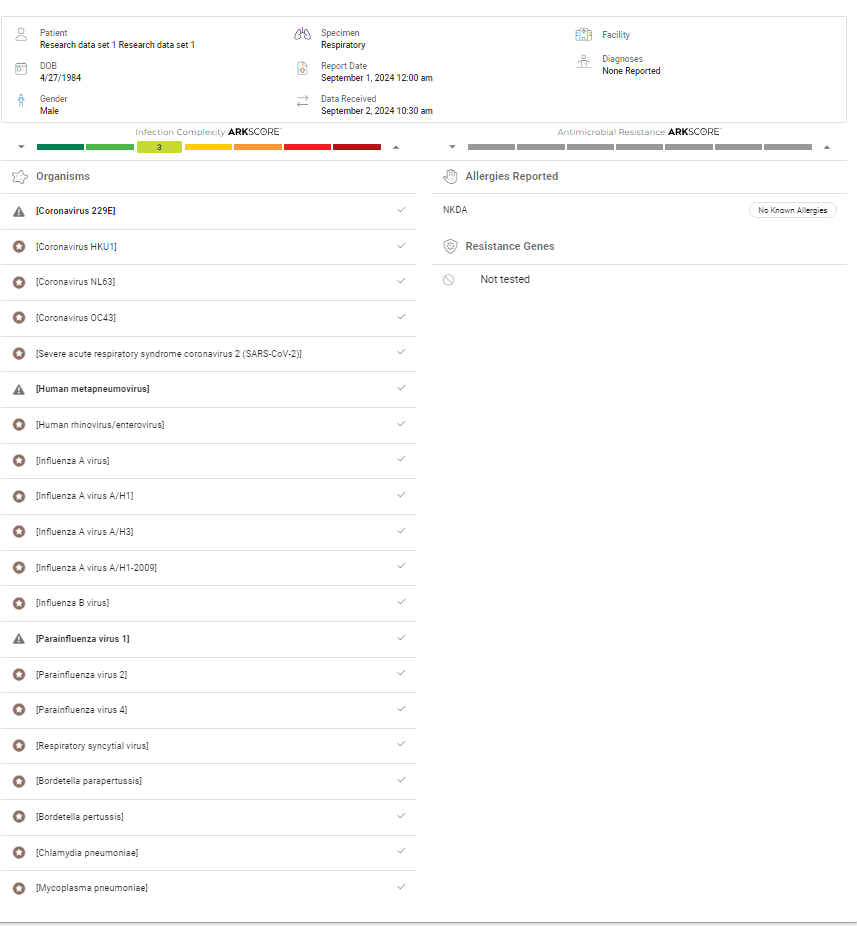

Supplement: Supplementary file 1 [file life-15-01123-s001.zip › Supllemnt 3 Validation study/K fold round 1/K fold test data set 1.PNG]

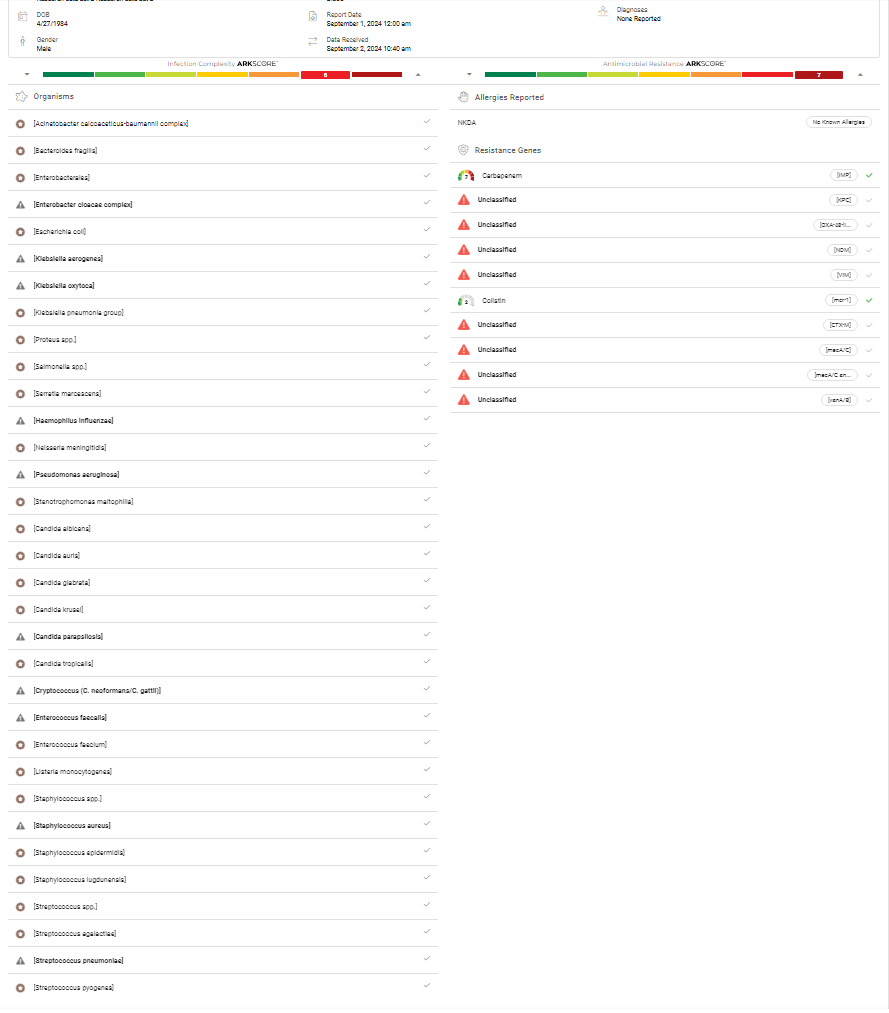

Supplement: Supplementary file 1 [file life-15-01123-s001.zip › Supllemnt 3 Validation study/K fold round 1/K fold testing data set 2.PNG]

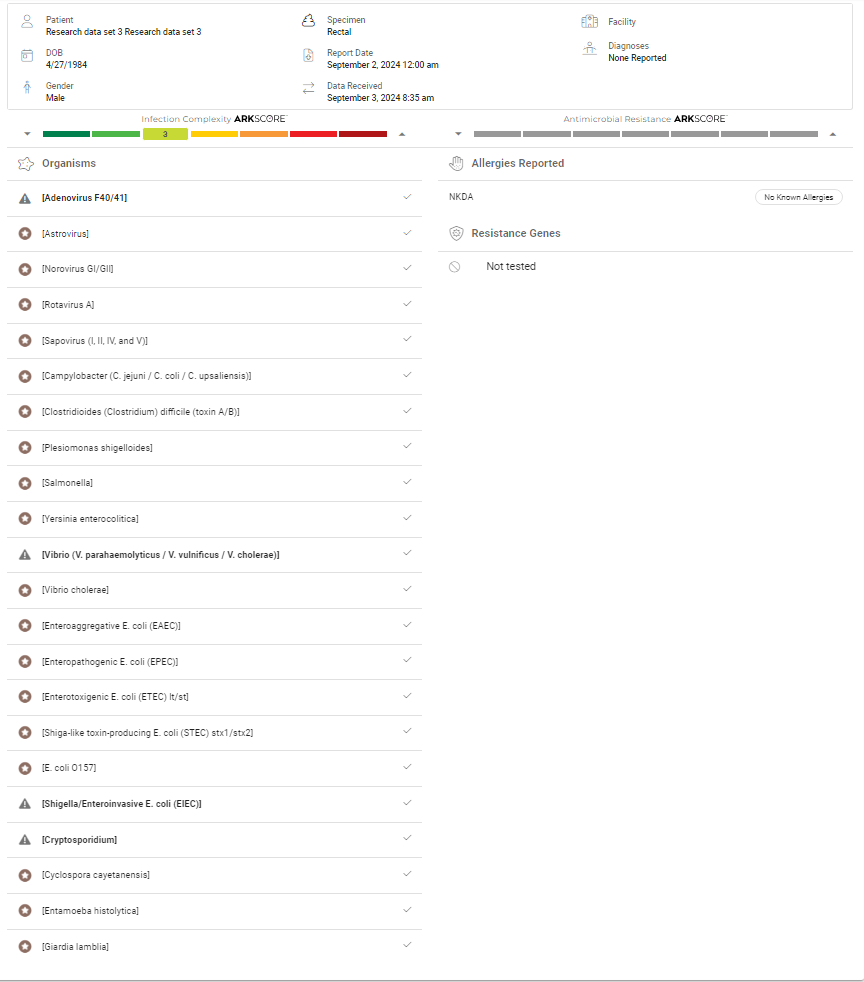

Supplement: Supplementary file 1 [file life-15-01123-s001.zip › Supllemnt 3 Validation study/K fold round 1/K fold testing Data set 3.PNG]

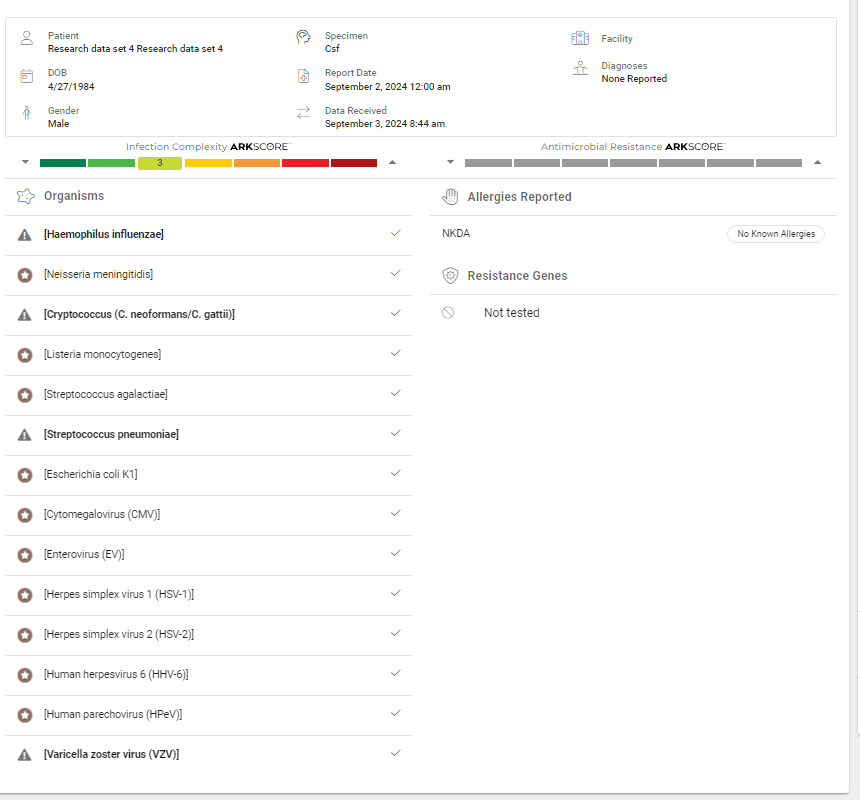

Supplement: Supplementary file 1 [file life-15-01123-s001.zip › Supllemnt 3 Validation study/K fold round 1/K fold testing data set 4.PNG]

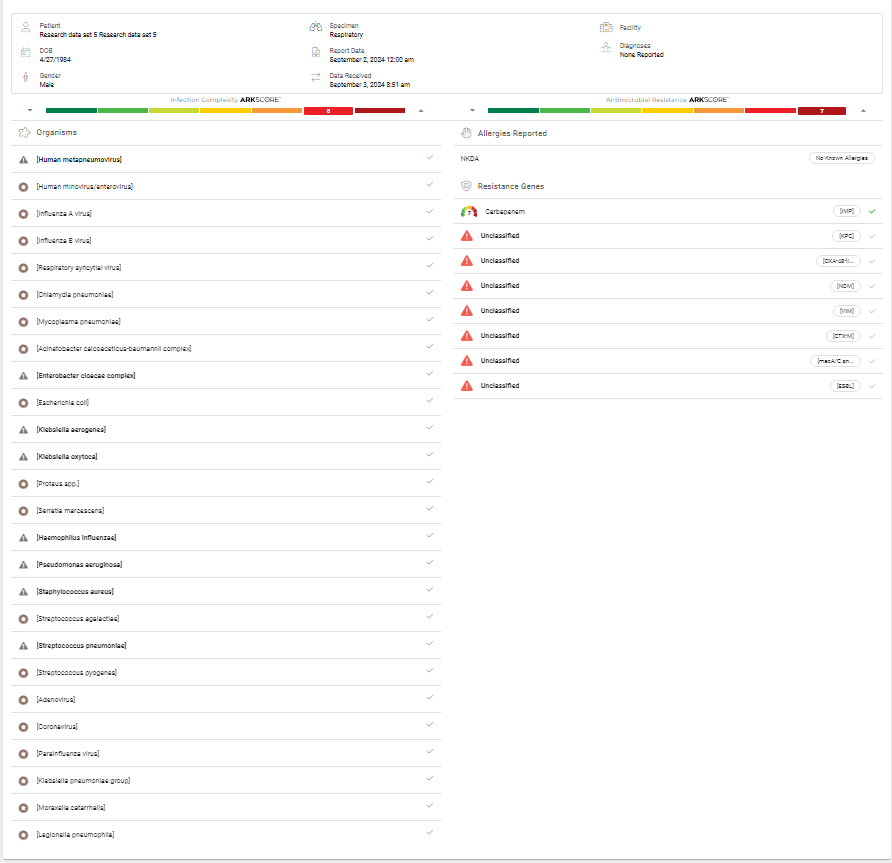

Supplement: Supplementary file 1 [file life-15-01123-s001.zip › Supllemnt 3 Validation study/K fold round 1/K fold testing Data set 5.PNG]

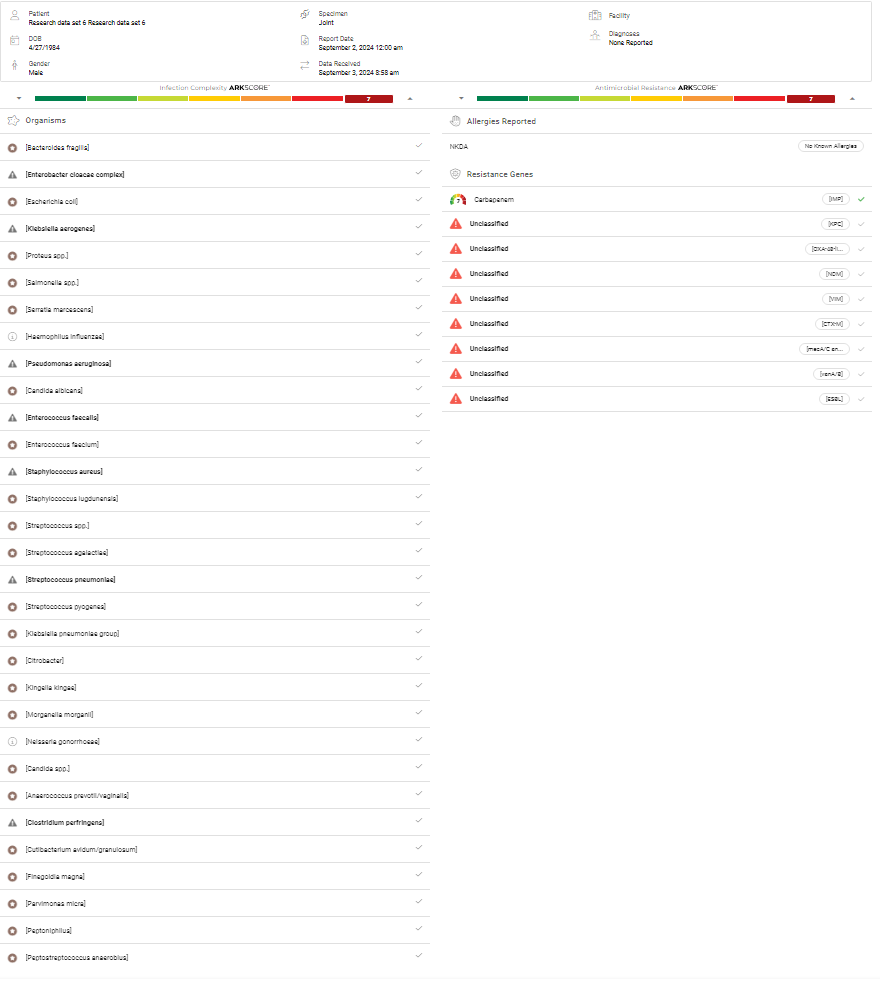

Supplement: Supplementary file 1 [file life-15-01123-s001.zip › Supllemnt 3 Validation study/K fold round 1/K fold testing data set 6.PNG]

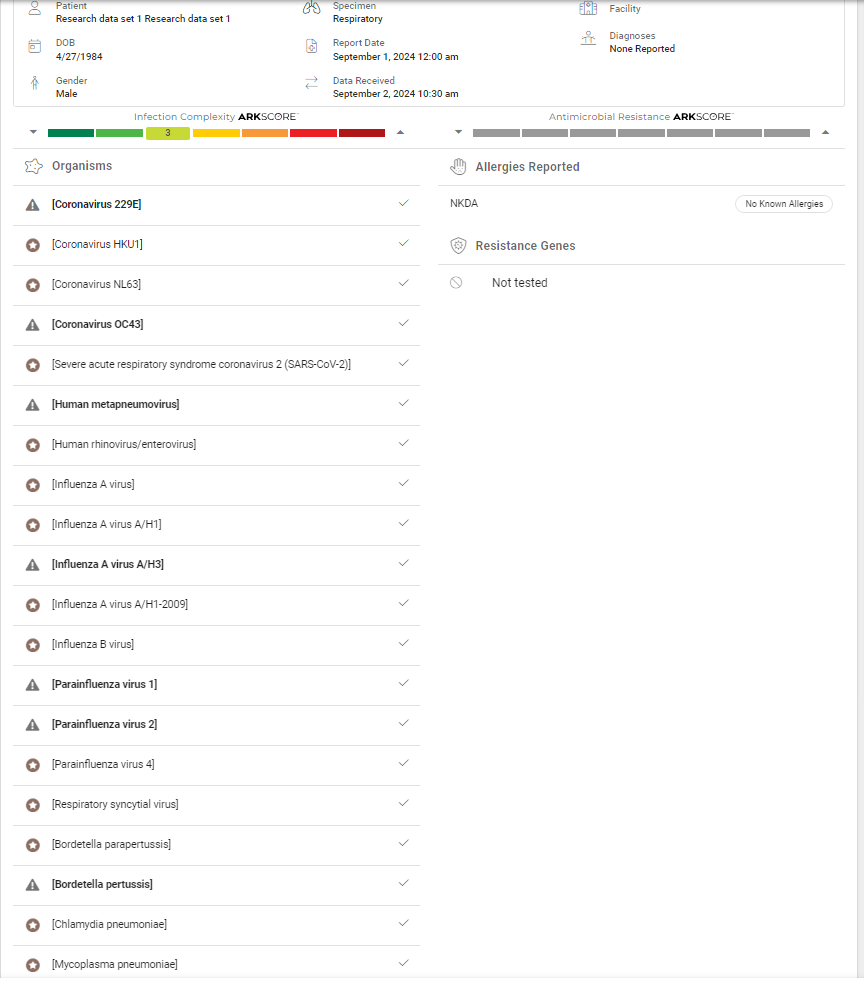

Supplement: Supplementary file 1 [file life-15-01123-s001.zip › Supllemnt 3 Validation study/K fold round 2/K fold data set 1 round 2.PNG]

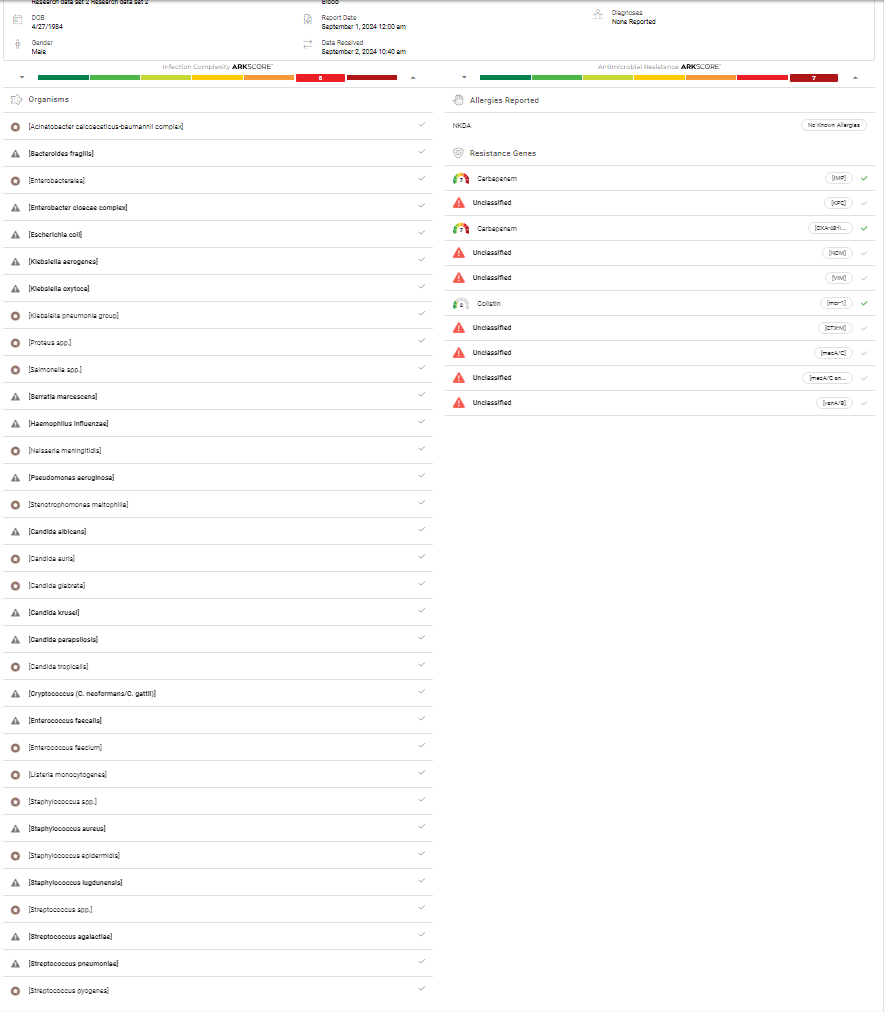

Supplement: Supplementary file 1 [file life-15-01123-s001.zip › Supllemnt 3 Validation study/K fold round 2/K fold data set 2 round 2.PNG]

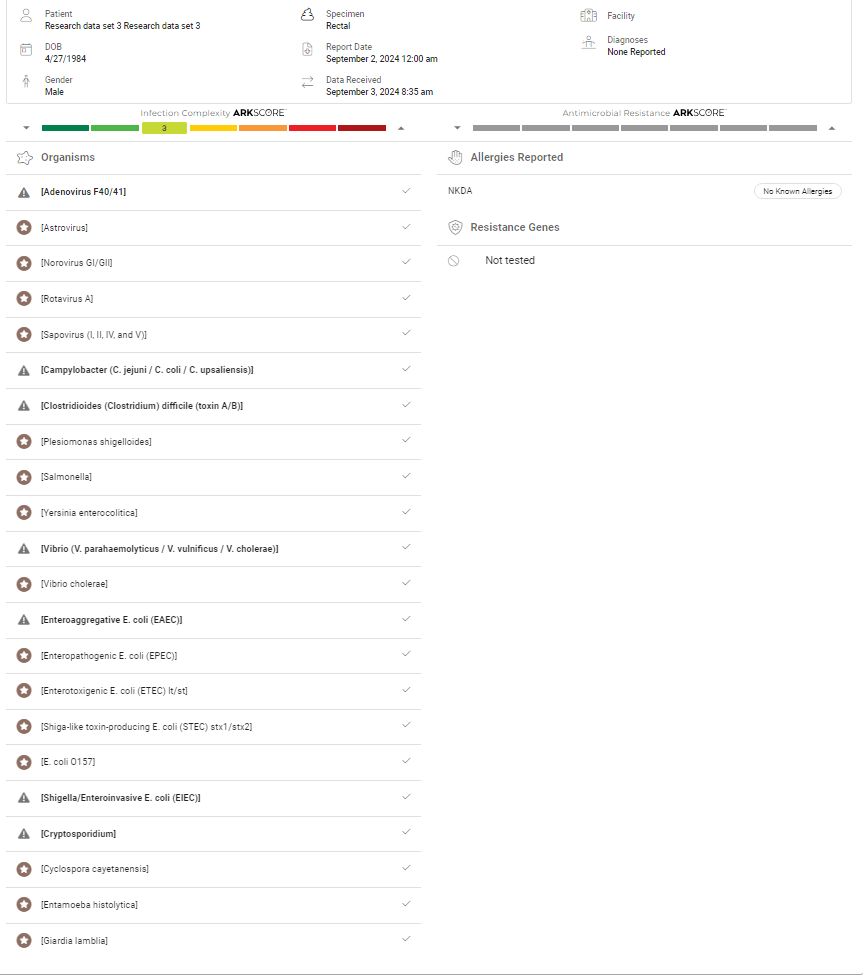

Supplement: Supplementary file 1 [file life-15-01123-s001.zip › Supllemnt 3 Validation study/K fold round 2/K fold data set 3 round 2.PNG]

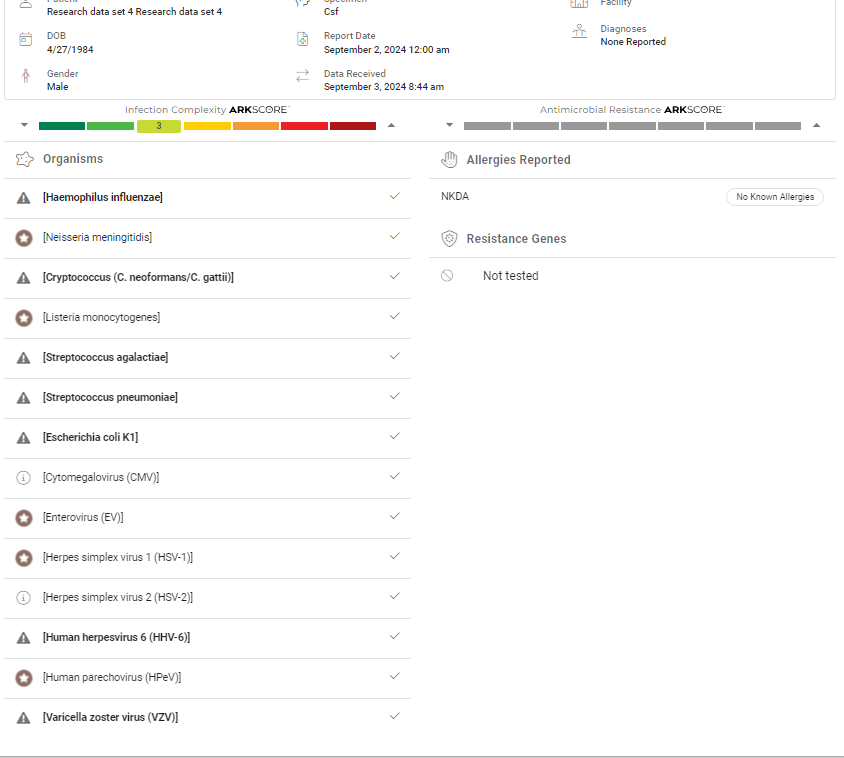

Supplement: Supplementary file 1 [file life-15-01123-s001.zip › Supllemnt 3 Validation study/K fold round 2/K fold data set 4 round 2.PNG]

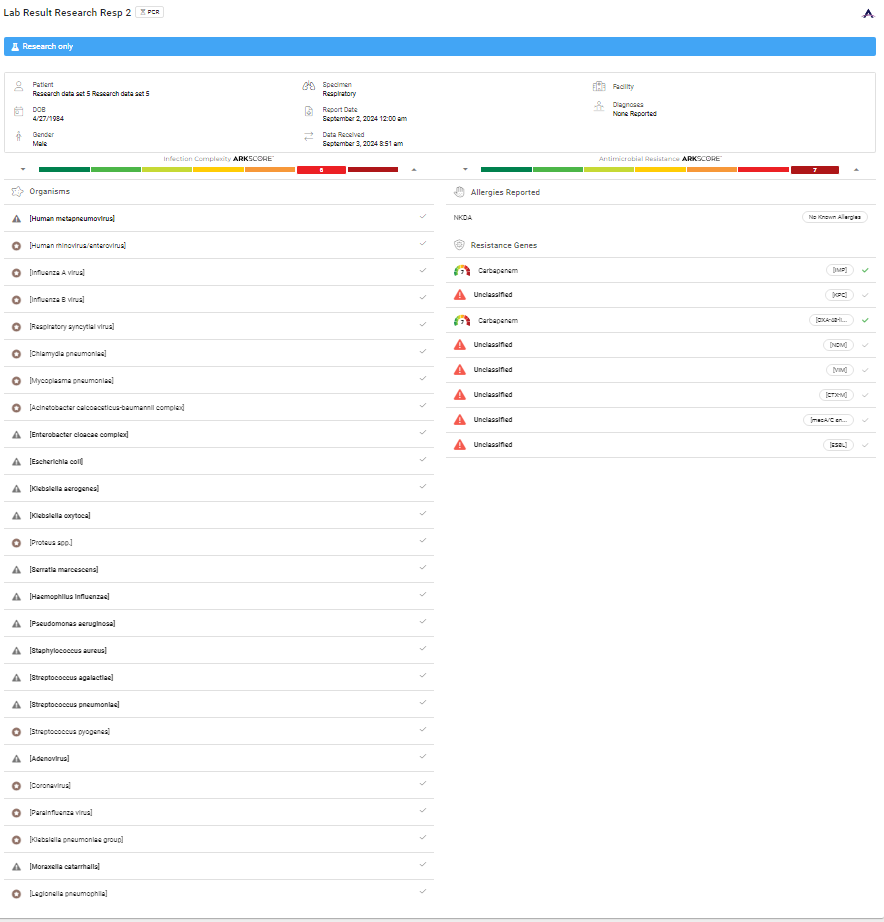

Supplement: Supplementary file 1 [file life-15-01123-s001.zip › Supllemnt 3 Validation study/K fold round 2/K fold data set 5 round 2.PNG]

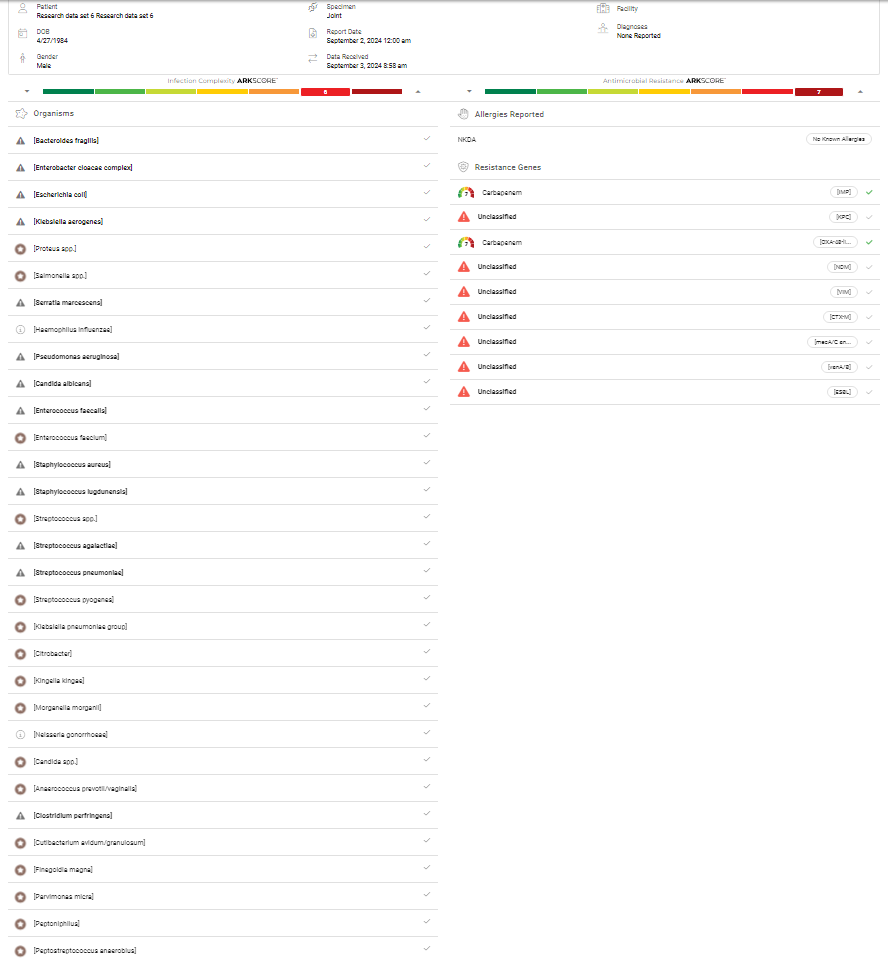

Supplement: Supplementary file 1 [file life-15-01123-s001.zip › Supllemnt 3 Validation study/K fold round 2/K fold data set 6 round 2.PNG]

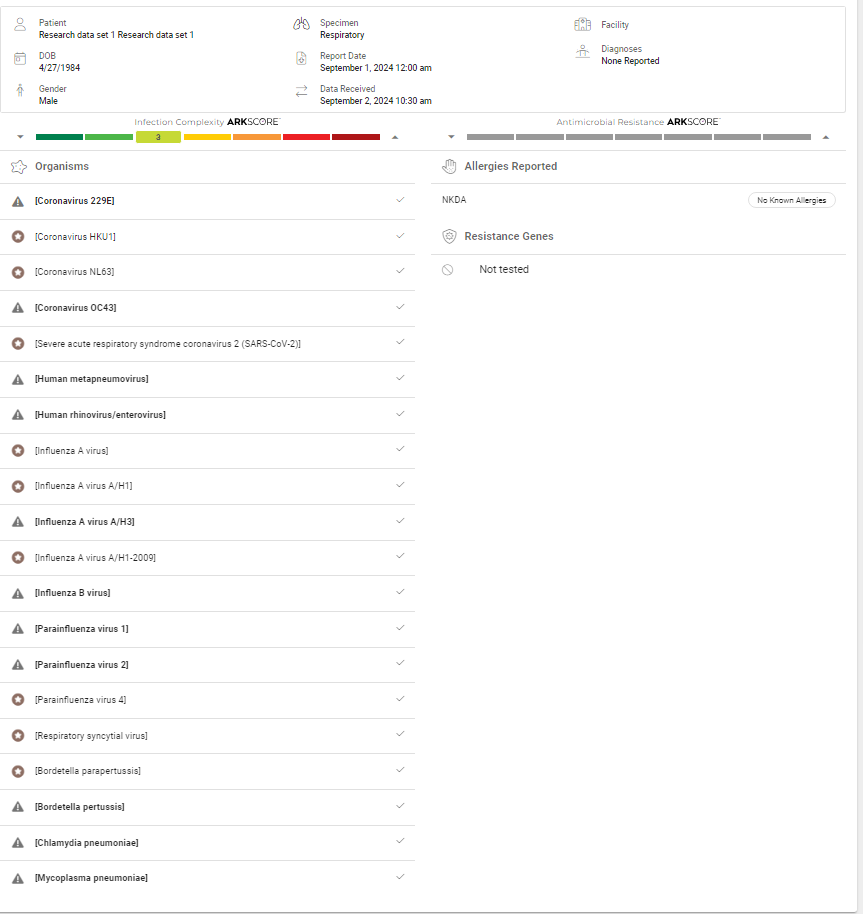

Supplement: Supplementary file 1 [file life-15-01123-s001.zip › Supllemnt 3 Validation study/K fold round 3/K fold data set 1 round 3.PNG]

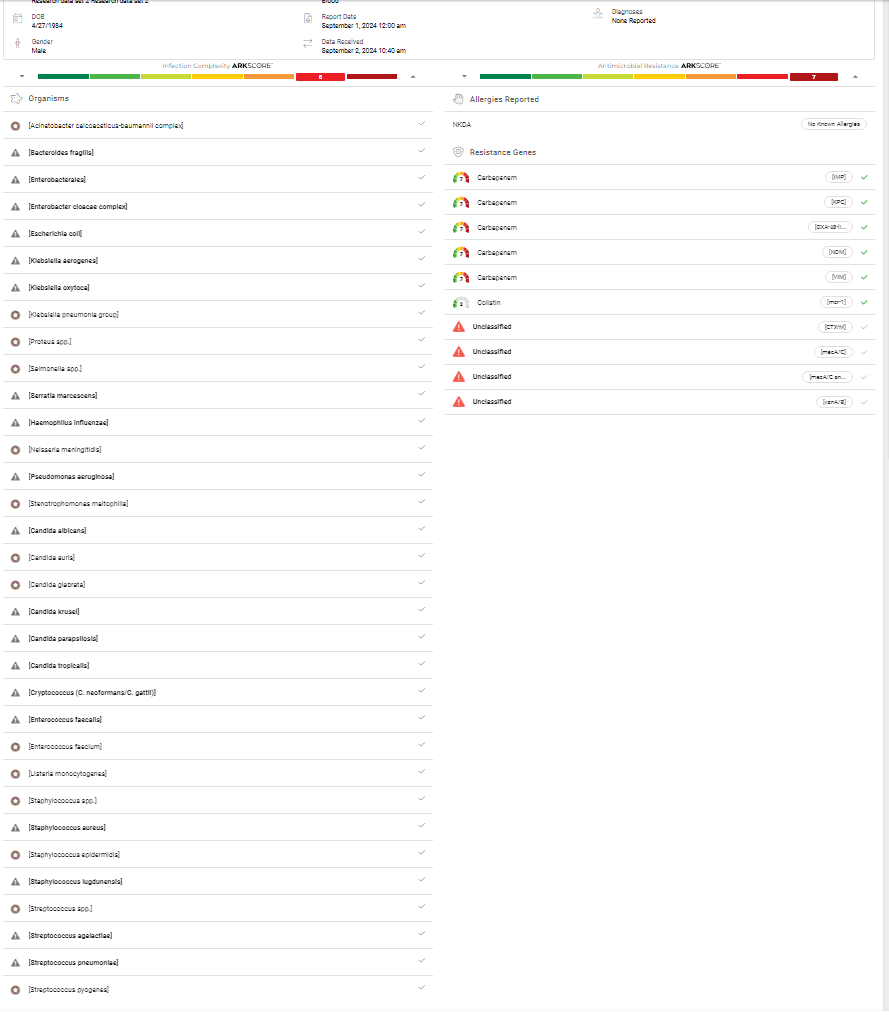

Supplement: Supplementary file 1 [file life-15-01123-s001.zip › Supllemnt 3 Validation study/K fold round 3/K fold data set 2 round 3.PNG]

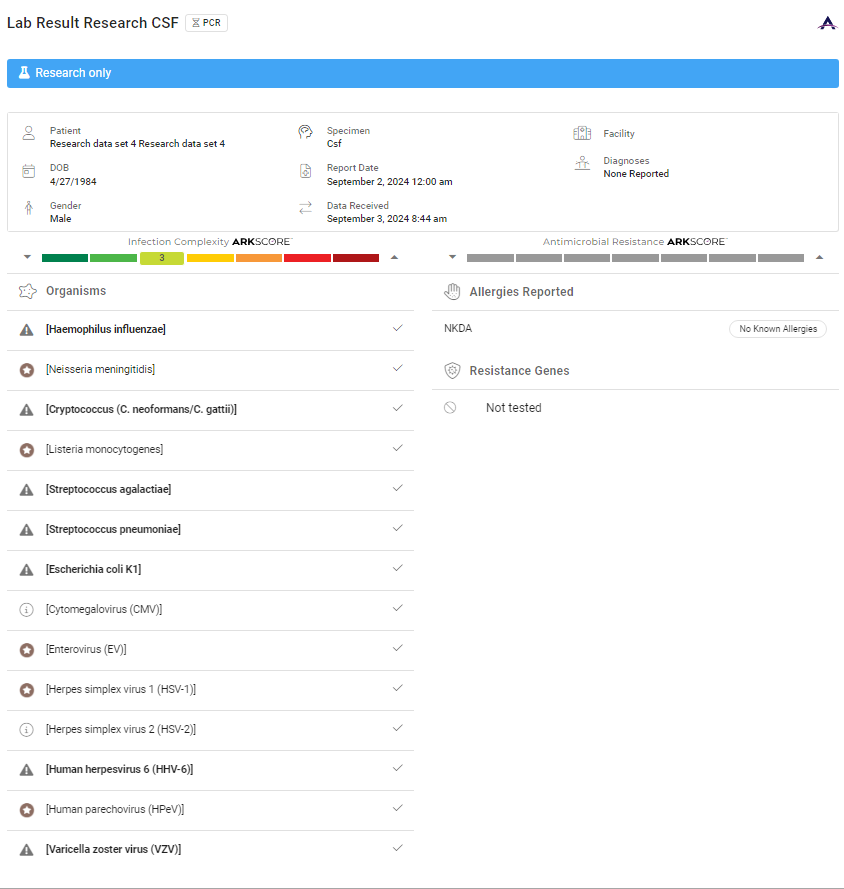

Supplement: Supplementary file 1 [file life-15-01123-s001.zip › Supllemnt 3 Validation study/K fold round 3/K fold data set 4 round 3.PNG]

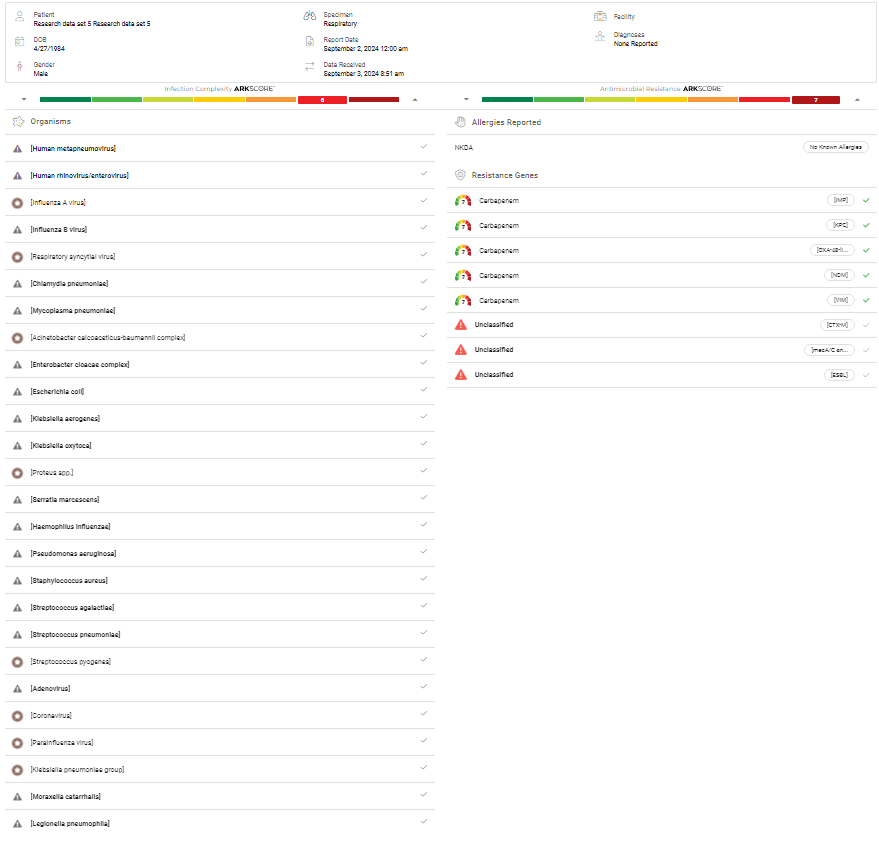

Supplement: Supplementary file 1 [file life-15-01123-s001.zip › Supllemnt 3 Validation study/K fold round 3/K fold data set 5 round 3.PNG]

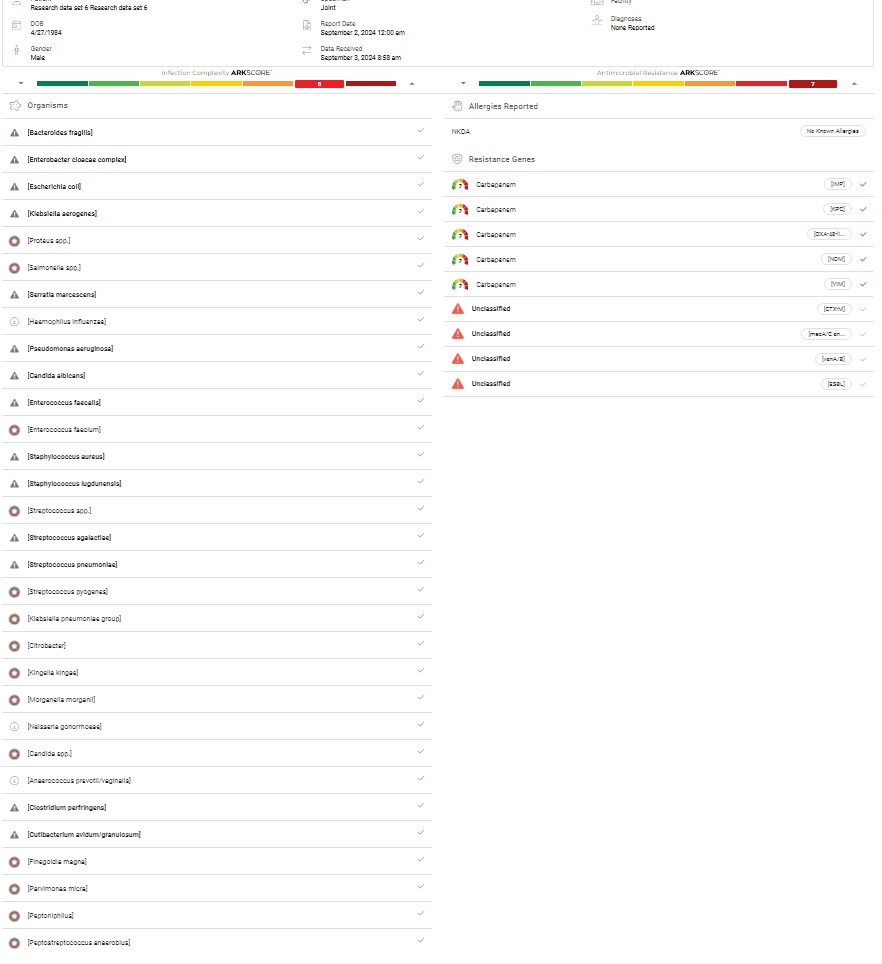

Supplement: Supplementary file 1 [file life-15-01123-s001.zip › Supllemnt 3 Validation study/K fold round 3/K fold data set 6 round 3.PNG]

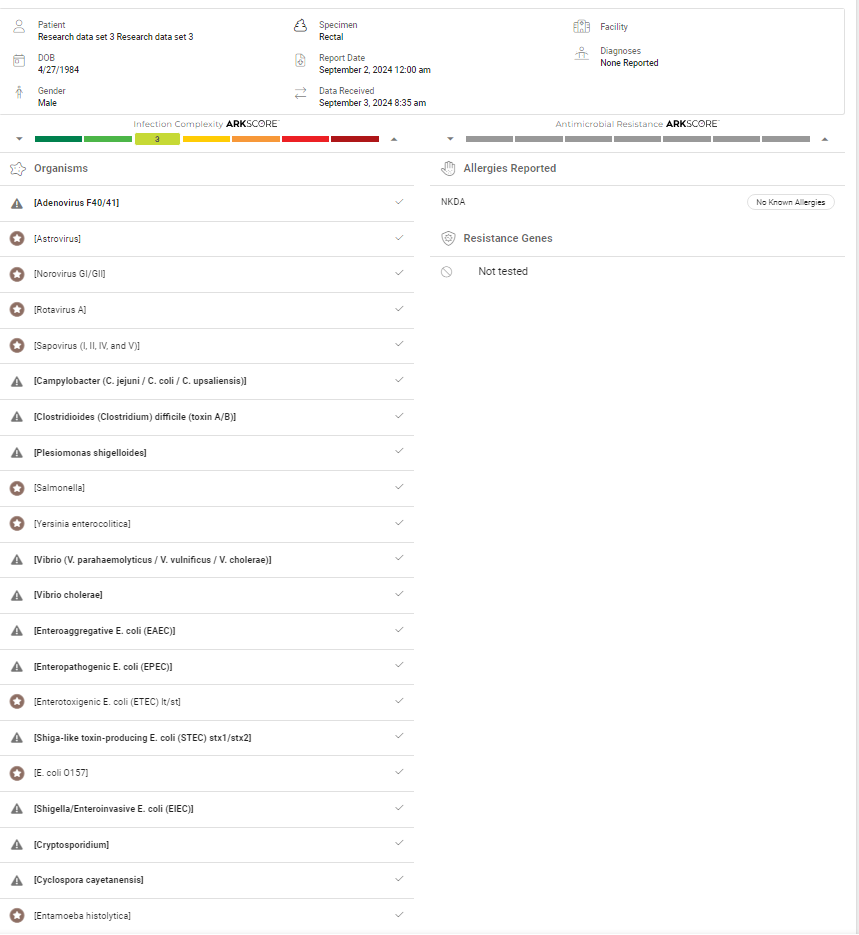

Supplement: Supplementary file 1 [file life-15-01123-s001.zip › Supllemnt 3 Validation study/K fold round 3/K fold data set round 3.PNG]

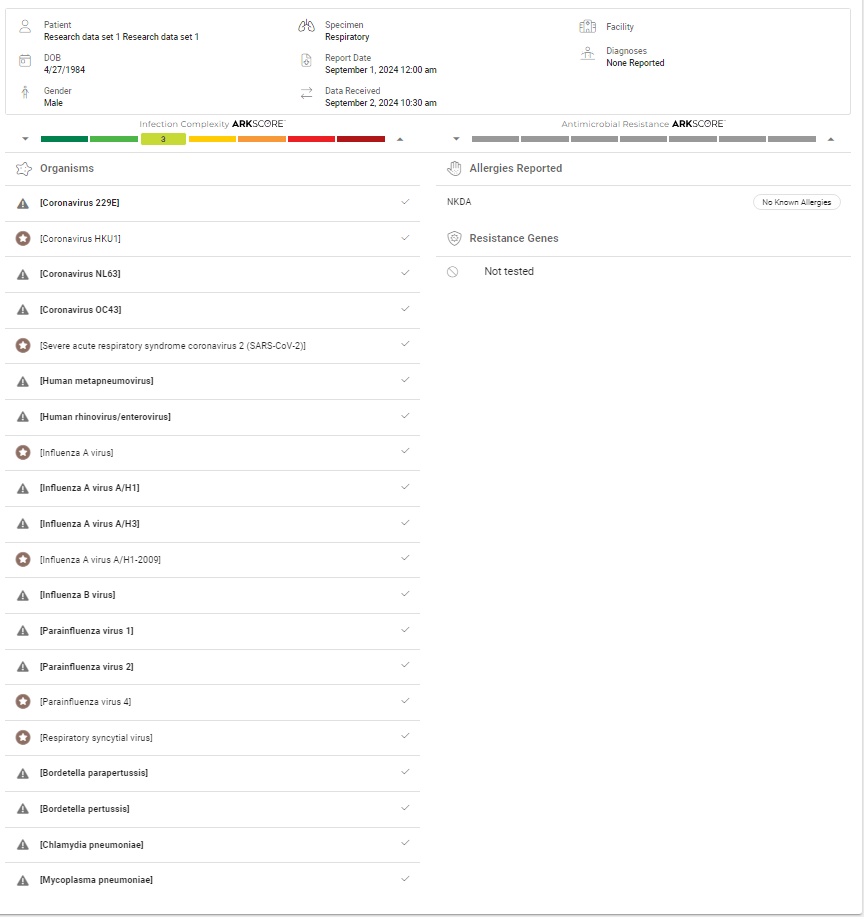

Supplement: Supplementary file 1 [file life-15-01123-s001.zip › Supllemnt 3 Validation study/K fold round 3/K fold round 4/K fold data set 1 round 4.PNG]

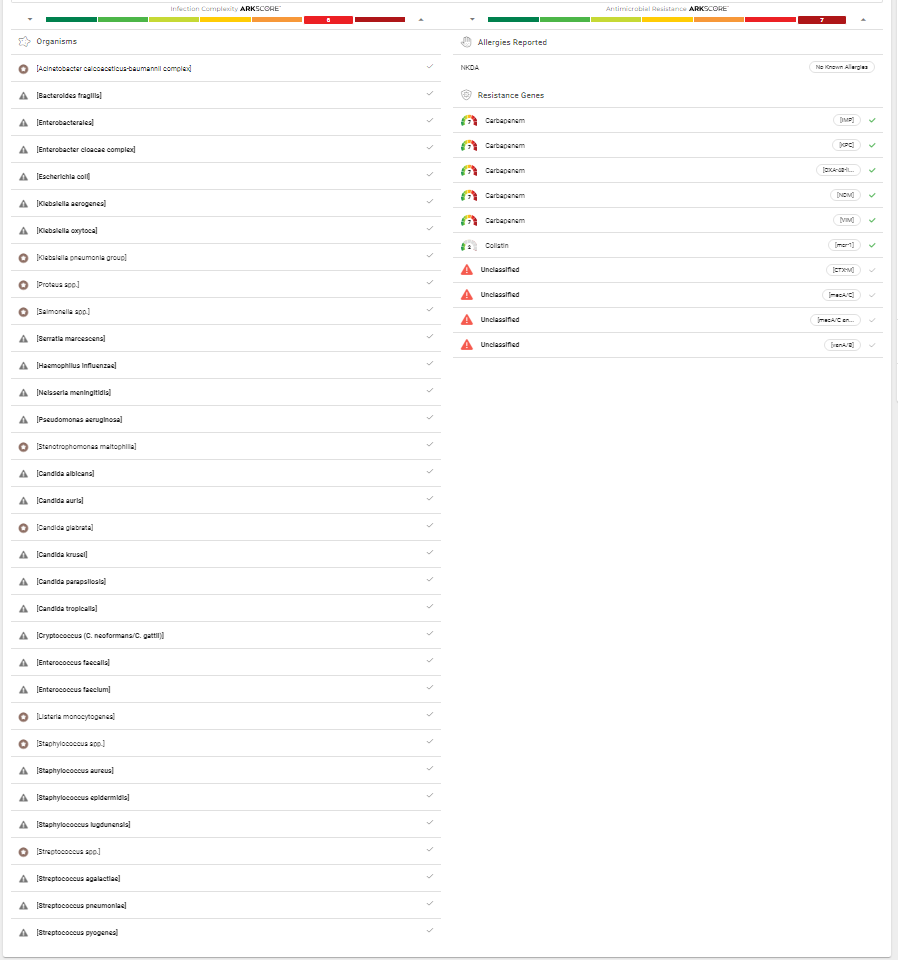

Supplement: Supplementary file 1 [file life-15-01123-s001.zip › Supllemnt 3 Validation study/K fold round 3/K fold round 4/K fold data set 2 round 4.PNG]

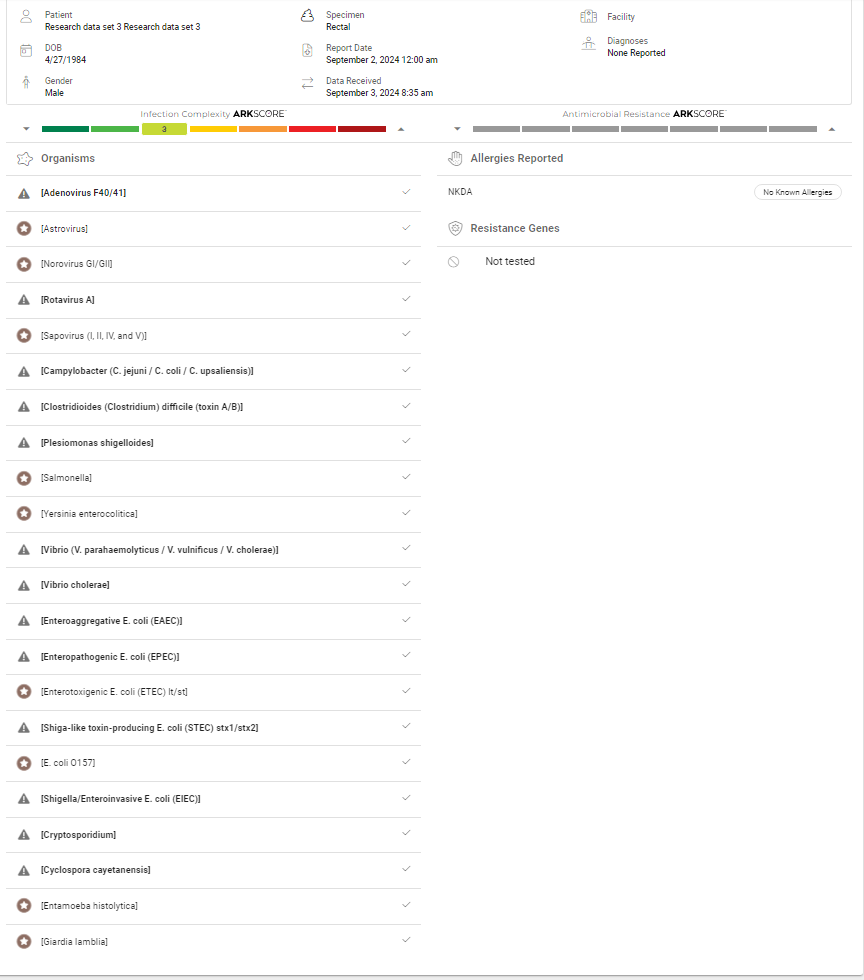

Supplement: Supplementary file 1 [file life-15-01123-s001.zip › Supllemnt 3 Validation study/K fold round 3/K fold round 4/k fold data set 3 round 4.PNG]

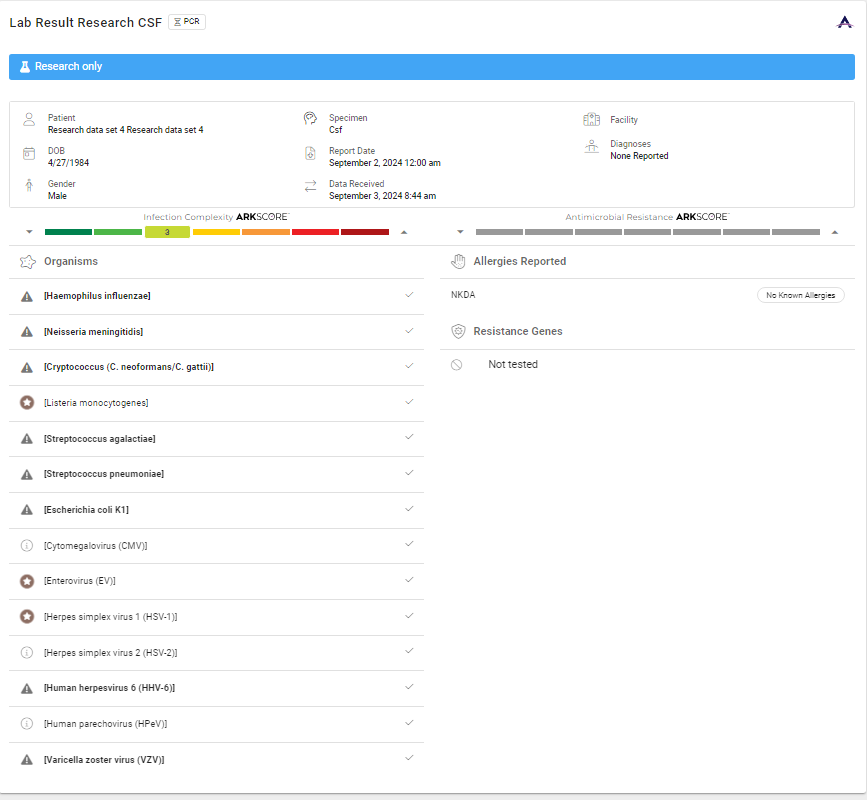

Supplement: Supplementary file 1 [file life-15-01123-s001.zip › Supllemnt 3 Validation study/K fold round 3/K fold round 4/K fold data set 4 round 4.PNG]

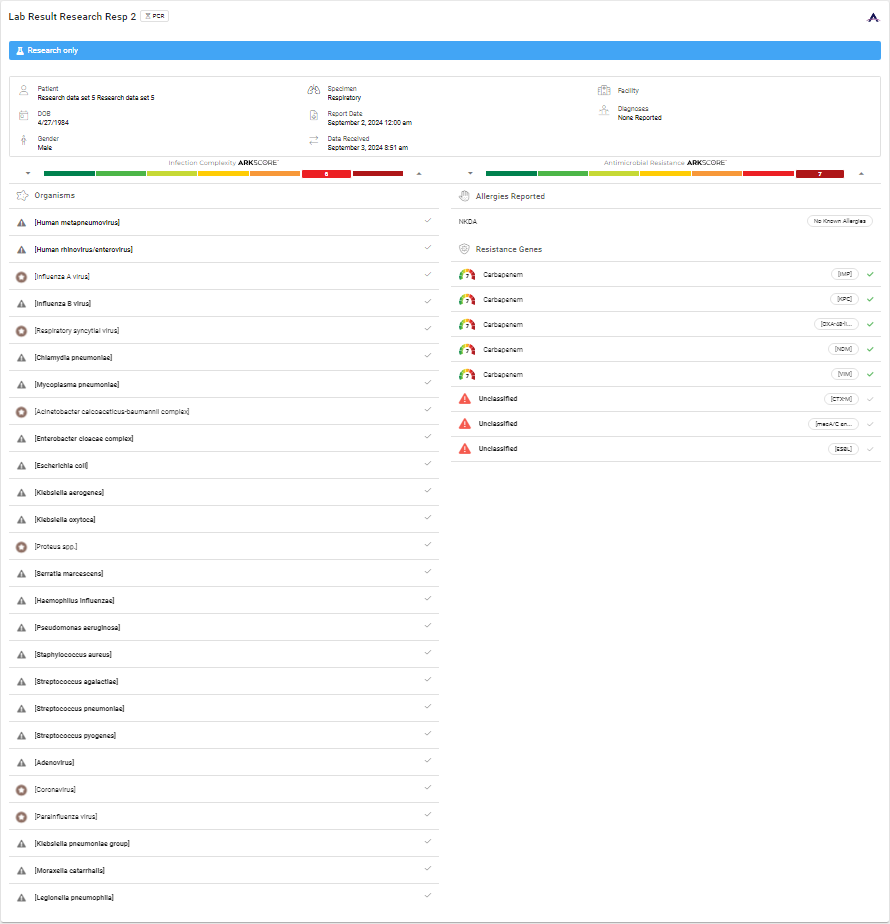

Supplement: Supplementary file 1 [file life-15-01123-s001.zip › Supllemnt 3 Validation study/K fold round 3/K fold round 4/K fold data set 5 round 4.PNG]

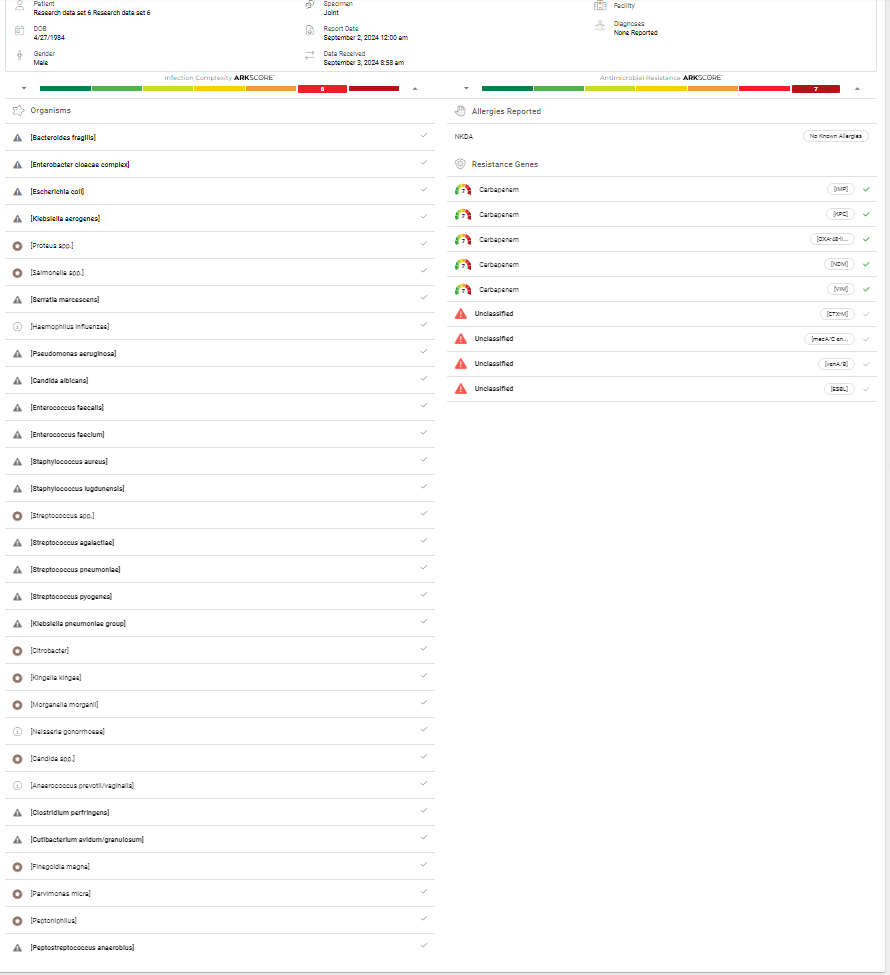

Supplement: Supplementary file 1 [file life-15-01123-s001.zip › Supllemnt 3 Validation study/K fold round 3/K fold round 4/K fold data set 6 round 4.PNG]

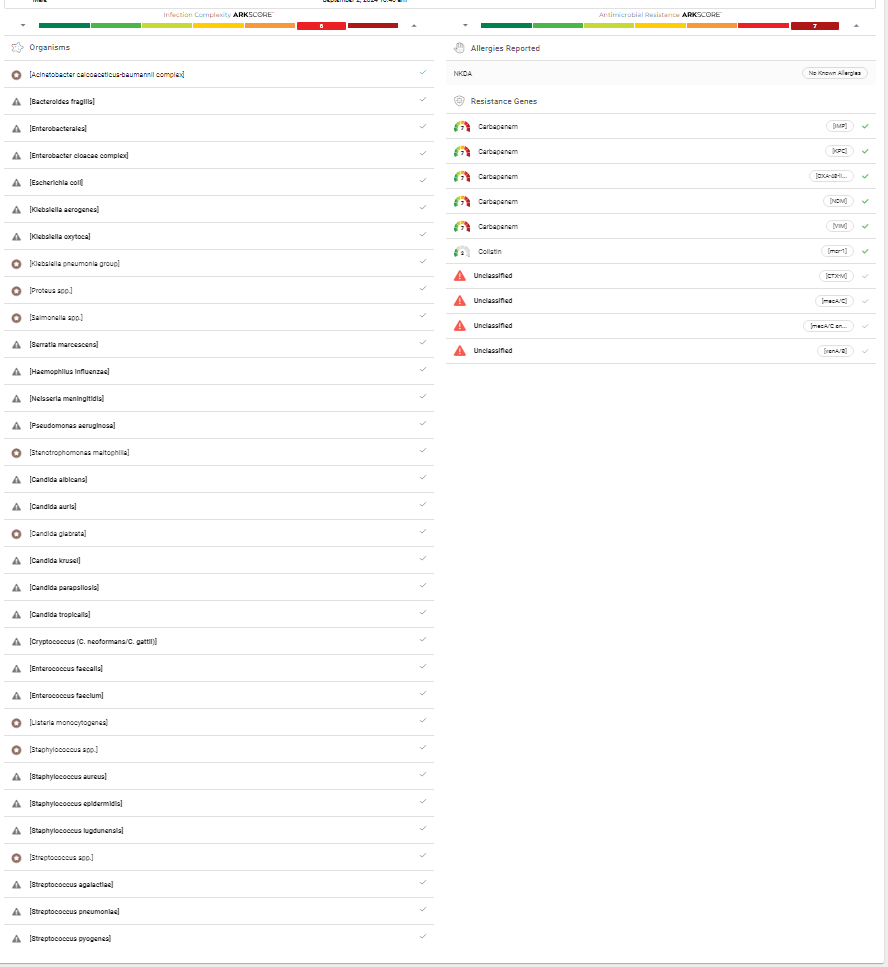

Supplement: Supplementary file 1 [file life-15-01123-s001.zip › Supllemnt 3 Validation study/K fold round 3/K fold round 4/K fold data set round 4.PNG]

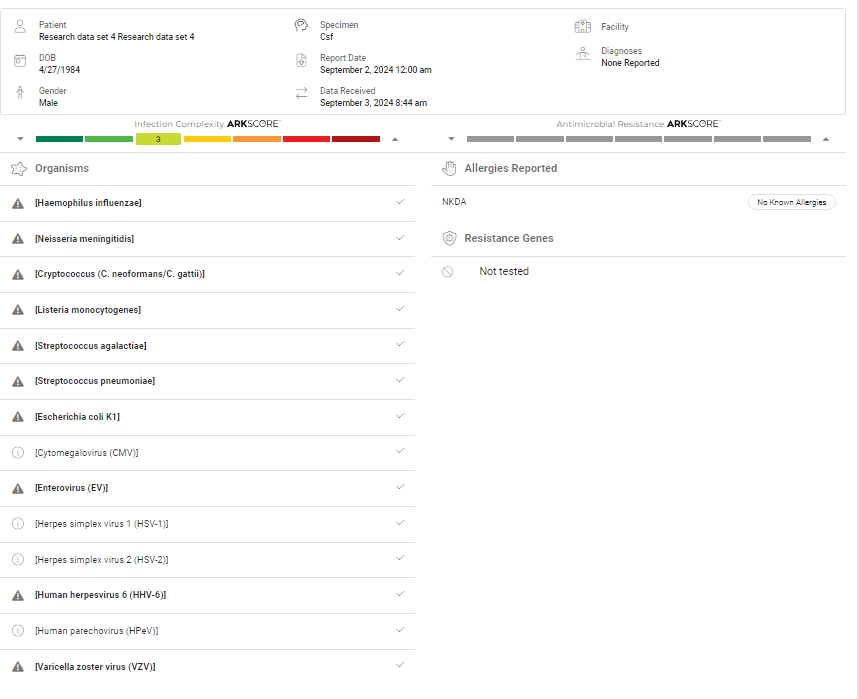

Supplement: Supplementary file 1 [file life-15-01123-s001.zip › Supllemnt 3 Validation study/K fold round 5/K fold data se 4 round 4.PNG]

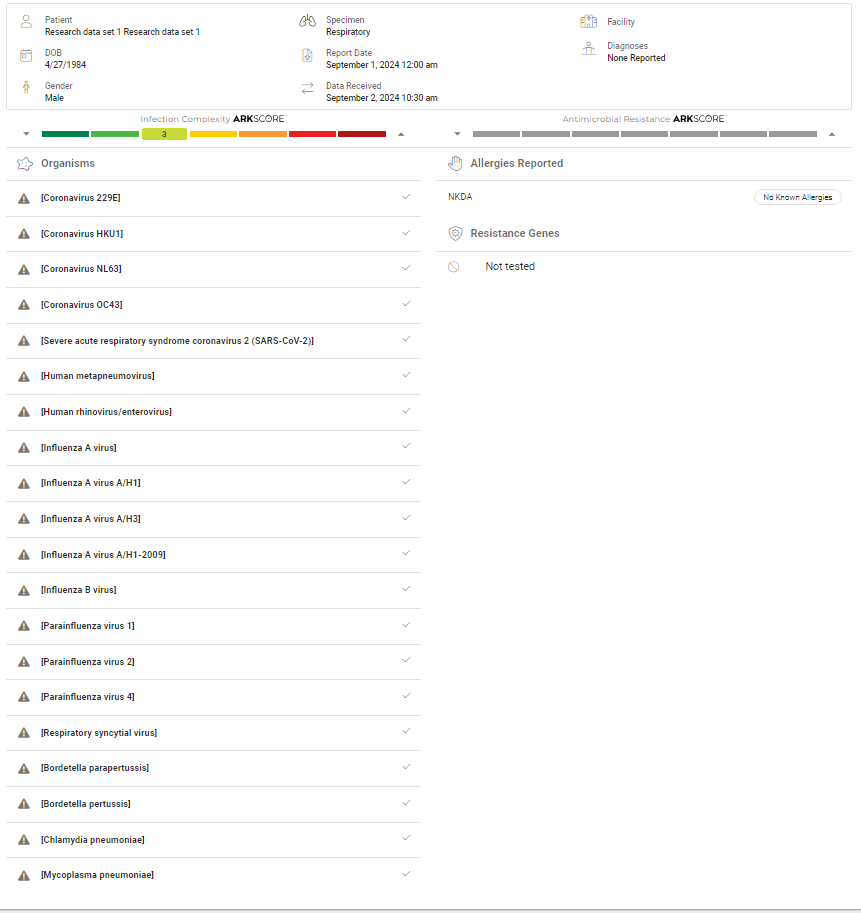

Supplement: Supplementary file 1 [file life-15-01123-s001.zip › Supllemnt 3 Validation study/K fold round 5/K fold data set 1 round 5.PNG]

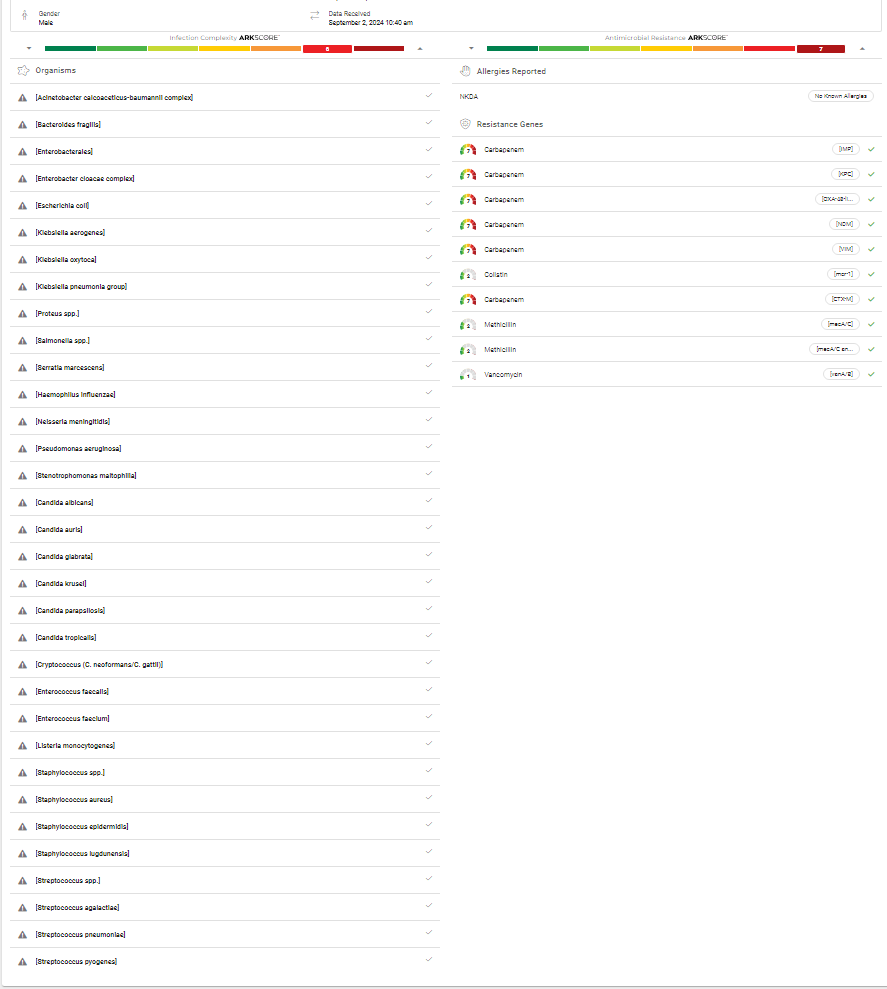

Supplement: Supplementary file 1 [file life-15-01123-s001.zip › Supllemnt 3 Validation study/K fold round 5/K fold data set 2 round 5.PNG]

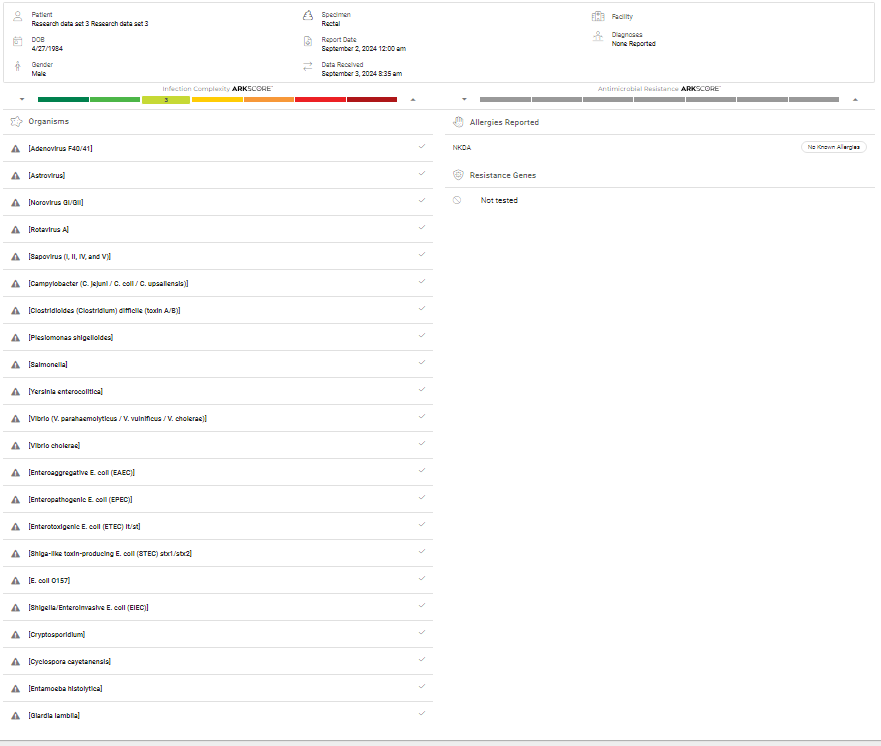

Supplement: Supplementary file 1 [file life-15-01123-s001.zip › Supllemnt 3 Validation study/K fold round 5/K fold data set 3 round 5.PNG]

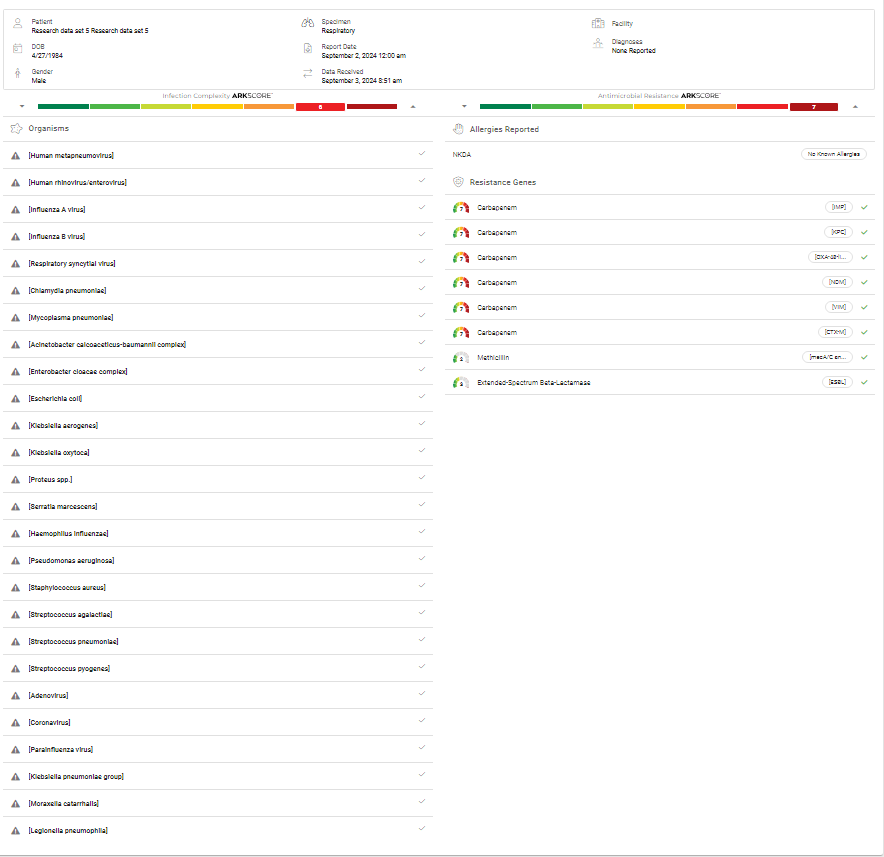

Supplement: Supplementary file 1 [file life-15-01123-s001.zip › Supllemnt 3 Validation study/K fold round 5/K fold data set 5 round 5.PNG]

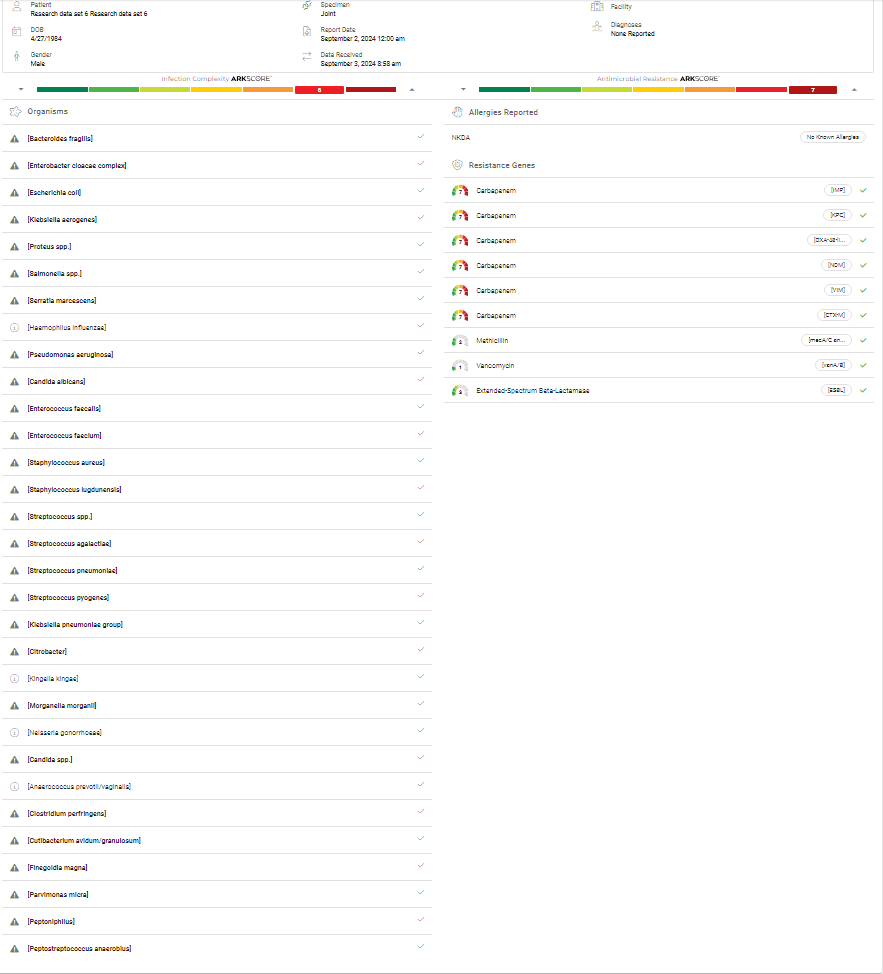

Supplement: Supplementary file 1 [file life-15-01123-s001.zip › Supllemnt 3 Validation study/K fold round 5/K fold data set 6 round 5.PNG]
